# Norbergenin prevents LPS-induced inflammatory responses in macrophages through inhibiting NFκB, MAPK and STAT3 activation and blocking metabolic reprogramming

**DOI:** 10.3389/fimmu.2023.1117638

**Published:** 2023-05-12

**Authors:** Wan Li, Zhengnan Cai, Florian Schindler, Sheyda Bahiraii, Martin Brenner, Elke H. Heiss, Wolfram Weckwerth

**Affiliations:** ^1^ Molecular Systems Biology (MOSYS), Department of Functional and Evolutionary Ecology, University of Vienna, Vienna, Austria; ^2^ Vienna Doctoral School of Ecology and Evolution, University of Vienna, Vienna, Austria; ^3^ Vienna Doctoral School of Pharmaceutical, Nutritional and Sports Sciences, University of Vienna, Vienna, Austria; ^4^ Department of Pharmaceutical Sciences, University of Vienna, Vienna, Austria; ^5^ Vienna Metabolomics Center (VIME), University of Vienna, Vienna, Austria

**Keywords:** norbergenin, anti-inflammation, proteomics, metabolomics, macrophages, NFκB signaling pathway, TLR - toll-like receptor, MAPK pathways

## Abstract

Inflammation is thought to be a key cause of many chronic diseases and cancer. However, current therapeutic agents to control inflammation have limited long-term use potential due to various side-effects. This study aimed to examine the preventive effects of norbergenin, a constituent of traditional anti-inflammatory recipes, on LPS-induced proinflammatory signaling in macrophages and elucidate the underlying mechanisms by integrative metabolomics and shotgun label-free quantitative proteomics platforms. Using high-resolution mass spectrometry, we identified and quantified nearly 3000 proteins across all samples in each dataset. To interpret these datasets, we exploited the differentially expressed proteins and conducted statistical analyses. Accordingly, we found that LPS-induced production of NO, IL1β, TNFα, IL6 and iNOS in macrophages was alleviated by norbergenin *via* suppressed activation of TLR2 mediated NFκB, MAPKs and STAT3 signaling pathways. In addition, norbergenin was capable of overcoming LPS-triggered metabolic reprogramming in macrophages and restrained the facilitated glycolysis, promoted OXPHOS, and restored the aberrant metabolites within the TCA cycle. This is linked to its modulation of metabolic enzymes to support its anti-inflammatory activity. Thus, our results uncover that norbergenin regulates inflammatory signaling cascades and metabolic reprogramming in LPS stimulated macrophages to exert its anti-inflammatory potential.

## Introduction

Macrophages are the central component of the innate immune system. They sense pathogens and rapidly promote an inflammatory phenotype to limit damage and support tissue homeostasis (1, 2). Inflammation is a complex defense process involving extensive signaling-dependent changes in gene expression (3, 4). This initially requires the engagement of Toll-like receptors (TLRs), which subsequently trigger the downstream activation of nuclear factor kappa B (NFκB) and mitogen-activated protein kinases (MAPKs, ERK, JNK and p38), which mediate fundamental biological processes and cellular response to external stimuli (5, 6). These events ultimately lead to production of various effectors, including inflammatory cytokines (IL1β, IL6 and TNFα) and nitric oxide synthase 2 (Nos2) eventually promoting the development of chronic inflammation (7, 8). Inflammation has critical protective functions, however, exacerbated and uncontrolled inflammatory processes can contribute to the pathology and progression of many chronic diseases, like diabetes, heart disease and cancer (9). Modulation of inflammation to properly sustain balance between its benefits and detriments offers intriguing therapeutical strategies for these diseases (1, 3). Lipopolysaccharides (LPS) are endotoxin molecules that derive from the cell wall of all gram-negative bacteria. Its administration to cells or animals induces strong immune responses. Therefore, LPS is commonly used as a stimulus to develop and study inflammation.

It has been reported that inflammatory macrophage activation by LPS is always accompanied by specific metabolic changes (10, 11). These alterations provide essential metabolic precursors for biosynthesis and energy for conforming to cellular functions. For example, LPS-induced macrophages display an enhanced glycolytic metabolism as well as impaired oxidative phosphorylation (OXPHOS). This resembles observations in cancer cells known as “Warburg effect”, along with high concentrations of lactate and pyruvate (12, 13). In addition, disruption of tricarboxylic acid (TCA) cycle at isocitrate dehydrogenase 1 (IDH1) and succinate dehydrogenase (SDH) is another metabolic hallmark of inflammatory macrophages with concomitant accumulation of citrate and succinate, which are well known as pro-inflammatory signals (14, 15). Moreover, LPS-induced macrophages display increased arginine and citrulline levels which are associated with elevated NO production by *Nos2*. Targeting these metabolic pathways holds great potential to alleviate inflammatory conditions (13, 16).

Conventional anti-inflammatory drugs such as aspirin and ibuprofen have been demonstrated to prevent pathological conditions associated with chronic inflammation (9). However, long-term use of these substances is linked to a high risk of adverse side effects, like hypertension, kidney damage and hepatic problems (17). Recently, there is a shift toward screening for healthy alternatives to controlling inflammation. Natural products from food and medicinal plants have similar anti-inflammatory activity to these drugs but far lower side effects (18, 19). Norbergenin, a polyphenolic gallic acid derivative, has been identified in many medicinal plants. Ethnobotanical use of norbergenin containing species has been reported in both Ayurveda and traditional Chinese medicine (20). In a previous study (21), norbergenin was proven to prevent arthritis through inhibiting pro-inflammatory cytokines IL6, IFNγ and TNFα as well as potentiating anti-inflammatory cytokines IL4 and IL5 in adjuvant-induced mice. It is well appreciated that infiltration of inflammatory cells and production of inflammatory mediators are responsible for developing and maintaining arthritis (22, 23). However, deep proteomics analysis of norbergenin on anti-inflammatory effects has not yet been performed, and the mechanisms of action underlying the anti-inflammatory effects of norbergenin remain largely unexplored. Inspired by above evidence, we set out to investigate the mechanism by which norbergenin impairs LPS-induced singaling in macrophages by using high-resolution liquid chromatography-mass spectrometry (LC-MS/MS)-based proteomics. To that end, we assembled control group, LPS group and LPS pretreated with norbergenin group. Analysis of macrophage samples revealed major functional differences between LPS pretreated with norbergenin and LPS alone, specifically involving NFκB signaling pathway and mitochondrial oxidative phosphorylation (OXPHOS). Our metabolomic analysis revealed that glycolysis and TCA cycle intermediates are major anti-inflammatory mediators of norbergenin in response to LPS.

## Materials and methods

### Reagents

Norbergenin (purity ≥ 98%) was obtained from Baoji Herbest Company (Baoji Herbest Bio-Tech Co., Ltd, Shanxi, China), LPS from *Escherichia coli* 055:B5 (Cat: L2880), Antimycin A (Cat: A8674), sodium pyruvate (Cat: 11360039), D-(+)-Glucose (Cat: G7021), D-glutamine (Cat: 158968) and 2-Deoxy-D-glucose (2-DG, Cat: D8375) were purchased from Sigma-Aldrich. SB203580 (Cat: HY-10256), U0126 (Cat: HY-12031), SP600125 (Cat: HY-12041), oligomycin (Cat: HY-N6782), rotenone (Cat: HY-B1756), FCCP (Cat: HY-50202), methylthiazolyldiphenyl-tetrazolium bromide (MTT) (Cat: HY15924) were obtained from MedChemExpress. Griess Reagent System (Cat: G2930) was obtained from Promega Corporation. Transfer membrane Immubilon-P PVDF (Cat: IPVH00010) was obtained from Millipore. All other reagents were of analytical grade and obtained from Sigma.

### Animals

6−8-week-old wild-type C57BL/6JRI mice were purchased from Janvier (France). The animals were kept in a pathogen-free environment. Every procedure was carried out under sterile conditions and according to the regulations of the Ethics Committee for the Care and Use of Laboratory Animals at the Medical University of Vienna.

### Cell culture and treatment

Bone marrow-derived macrophages (BMDMs) were prepared from C57BL/6JRI mice aged from 6 to 8 weeks as previously described (24). In brief, femurs and tibiae were flushed and bone marrow cells collected by centrifugation at 400 × g for 5 min at 4 °C. Then bone marrow cells were plated at non-tissue culture treated petri dishes in BMDMs differentiation media consisting of Dulbecco’s modified Eagle’s medium (DMEM)/high glucose (Lonza or Gibco), 10% heat-inactivated fetal bovine serum (FBS) (Gibco), 2 mM L-glutamine (Invitrogen), 100 U/mL penicillin (Invitrogen) and 100 μg/mL streptomycin (Invitrogen) and 20% filtered L929-conditioned supernatant. Plates were fed with fresh differentiation media on day 3 of differentiation. Until day 6 or 7, differentiated BMDMs were detached and then resuspended in media and plated on 6-well plates for different treatments.

Murine immortalized bone marrow-derived macrophages (iBMDMs) were kindly provided by Laszlo Nagy (Debrecen University, Hungary) and cultured in DMEM supplemented with 15% heat-inactivated FBS, 10% L929-conditioned supernatant, 2 mM L-glutamine, 100 U/mL penicillin and 100 μg/mL streptomycin.

RAW264.7 macrophages were obtained from American Type Culture Collection (ATCC, USA) and cultured in DMEM medium supplemented with 10% heat-inactivated FBS, 2 mM L-glutamine, 100 U/mL penicillin and 100 μg/mL streptomycin.

For treatment, BMDMs, iBMDMs and RAW264.7 macrophages were pretreated with or without norbergenin (5, 10, and 50 μM) for 1 h and then stimulated with LPS at a concentration of 200 ng/mL for 8 h.

### Cell viability

For cell viability assay, iBMDMs and BMDMs were seeded onto 96-well plates at 5 × 10^4^ cells/well for overnight. Then cells were treated with norbergenin at concentrations of 1, 5, 10 and 50 μM, respectively for 24 h. 5 μL of MTT (5 mg/mL) was added into the cells for another 4 h of incubation. Pure dimethyl sulfoxide (DMSO) (Cat: M81802) was used to dissolve the formazan crystals and then measured at 570 nm with a microplate reader. The values were analyzed for the cell viability.

### Nitric oxide (NO) production

iBMDMs (1 × 10^5^) were seeded in 96-well plates overnight and preincubated with norbergenin (5, 10 and 50 μM) for 1 h. Then cells were stimulated with LPS (200 ng/mL) for 24 h. NO production was determined by Griess reagent system based on manufacturer’s instructions.

### Enzyme-linked immunosorbent assay (ELISA)

After treatment, the concentration of TNFα in iBMDMs culture medium was measured by ELISA kit (Cat: RAB0477-1, Sigma) according to the manufacturer’s instruction. Absorbances were determined at a wavelength of 450 nm using a microplate reader (Thermo Fisher Scientific).

### RNA extraction and real-time qPCR

Total RNA was isolated using TRI reagent (Sigma) according to manufacturer’s protocols. RNA quantities were determined using the Qubit 4 Fluorometer and Qubit RNA Broad Range Assay Kits (Cat: Q10210, Invitrogen) following instructions as well as Nanodrop (Thermo Fisher Scientific). Reverse transcription of RNA was performed with the GoScript TM Reverse Transcription Kit (Promega) using 1 μg total RNA. Target mRNA levels were further assessed by the Luna Universal qPCR Master Mix (Cat: M3003E, New England Biolab) on a Bio-rad CFX96 real-time system. The quantification of mRNA expression of genes was analyzed by the comparative Ct (2-^ΔΔCt^) method. *Rps9* gene was taken as the internal control. Primer pair sequences were listed in supporting materials (**Table S3**).

### Western blotting

iBMDMs were cultured in 6-well plates at a density of 1 × 10^6^ cells/well overnight and then treated in DMEM medium with 3% FBS. After treatments, cells were lysed in cold RIPA lysis buffer containing protease inhibitor cocktails (Cat: 4693116001, Sigma) and phosphatase PhosStop EASYPack cocktails (Cat: 4906837001, Carl Roth) followed by sonication with a tip-probe sonicator (3 s of 1 s on and 1 s off at 70% output power) on ice. The concentrations of protein samples were determined by the BCA kit (Cat: 71285-3, Millipore). Equal amounts of proteins were loaded on and separated by 10% SDS-PAGE and transferred onto PVDF membranes. The membranes were blocked in 5% (w/v) low-fat milk for 1 h at room temperature and then incubated with primary antibodies. Afterwards, they were incubated with corresponding horseradish-peroxidase-conjugated (HPR) secondary antibodies. The protein bands were visualized by the ECL system (Cat: GERPN2232, Cytiva) and acquired using iBright FL1500 Imaging System (Thermo Fisher Scientific). The primary antibodies used were anti-NFκB (Cat: sc-8008), anti-p-NFκB (Cat: sc-136548), anti-IκBα (Cat: sc-1643), anti-p-IκBα (Cat: sc-8404), anti-p38 (Cat: sc-7972), anti-p-p38 (Cat: sc-166182), anti-HIF-1α (Cat: sc-13515), anti-STAT3 (Cat: sc-8019), anti-p-STAT3 (Cat: sc-8059), anti-TLR4 (Cat: sc-293072) obtained from Santa Cruz (Heidelberg, Germany), anti-IL1β (Cat: 12242S), anti-SAPK/JNK (Cat: 9252S), anti-p-SAPK/JNK (Cat: 4668S), anti-ERK (Cat: 4695S), anti-p-ERK (Cat: 9101S), anti-TLR2 (Cat: 13744S) obtained from Cell Signaling Technology (Danvers, USA). Anti-iNOS (Cat: 18985-1-AP) and α-Tubulin (Cat: 1224-1-AP), horseradish-peroxidase (HRP)-conjugated anti-Rabbit Ig(H + L) (Cat: SA00001-2), (HRP)-conjugated anti-Mouse Ig(H + L) (Cat: SA00001-1) were purchased from Proteintech (Munich, Germany).

### Seahorse extracellular flux analysis

iBMDMs were plated in 6-well plates at a density of 1 × 10^6^ cells per well. Following the treatment of cells as described before, cells were scraped and seeded onto a Cell-Tak (Cat: 354240, Corning)-precoated XF24e-cell culture plates for immediate adhesion with XF assay medium (Cat: 103575, Agilent Technologies). Before measurement, cells were placed in a CO_2_-free incubator at 37°C for 1 h. In advance, 1 mL calibrant solution was added to each well of a utility plate allowing for hydrating XF extracellular flux cartridge through overnight incubation in a CO_2_-free incubator at 37°C. According to the instruction of glycolysis stress assay from Agilent, a final injection of glucose (30 mM), oligomycin (30 μM) and 2-DG (50 mM) were added to each well. Real-time extracellular acidification (ECAR) was measured by the seahorse analyzer. For the mitochondrial stress assay, cells were incubated in XF assay medium supplied with 2 mM glutamine, 1 mM pyruvate and 25 mM glucose. Final concentrations of oligomycin (3 μM), FCCP (3 μM), and rotenone/antimycin A (0.5 μM) were injected into the ports of A, B, and C. Data were monitored as ECAR and OCR and analyzed by the Wave software from Agilent Technologies.

### siRNA transfection

RAW 264.7 macrophages were seeded onto 6-well plates at a density of 1 × 10^6^ cells/well. When reaching 50% confluence, cells were transfected with control siRNA or TLR2 siRNA using lipofectamine 3000 Transfection Reagent (Invitrogen). After 48 h transfection, the cells were washed with PBS gently and then pretreated with or without norbergenin (50 μM) for 1 h followed by stimulation with LPS (200 ng/mL) for another 8 h. After treatment, the cells were collected to analyze protein and mRNA expression. The TLR2 siRNA sequences were as follows: 5’-GGUUCCUUGUUUACUUUCATT-3’, 5’-UGAAAGUAAACAAGGAACCTT-3’.

### Proteome sample preparation

Proteomic analyses were performed according to established protocols with modifications (24). Briefly, iBMDMs were seeded in DMEM complete medium and allowed to attach for overnight. Cells were pretreated with norbergenin (50 μM) for 1 h followed by stimulation with LPS (200 ng/mL). After 8 h incubation, cells were washed and harvested in GdmCl buffer (6M guanidinium chloride (GdmCl), 100 mM Tris pH 8.5, 10 mM tris-(2-carboxyethyl)-phosphin-hydrochloride (TCEP), 40 mM 2-chloroacetamide (CAA)). Lysates were heated for 5 min at 95°C and sonicated with a tip–probe sonicator at 4°C (3 × 30 s of 1 s on and 1 s off at 80% output power). The protein concentration was determined by a BCA assay and adjusted to a concentration of 0.5 µg/µl. 50 µg of protein solution was diluted with 15% aqueous acetonitrile (ACN), and digested with 100:1 (protein:enzyme) LysC at 37°C for 2 h. Then, 10% aqueous ACN in 25 mM Tris (pH 8.5) was added to obtain a final concentration of 0.5 M GdmCl and a final volume of 1000 µL. Samples were incubated with trypsin 50:1 (protein:enzyme) overnight at 37°C. Digested peptides were acidified to a final concentration of 1% Trifluoroacetic acid (TFA). The peptides were then desalted with MonoSpin C18 columns (GL science) according to the manufacturer’s instruction. The peptides were eluted with 2 × 60 µl ACN and concentrated in a SpeedVac (Labogene) for 1 h at 30°C. Finally, they were reconstituted in MS buffer (2% ACN, 0.1% formic acid (FA)) for LC-MS/MS analysis. The experiment was performed in 4 biological replicates.

### LC-MS-based label-free proteomic analysis

LC-MS/MS runs were performed on the UltiMate 3000 RSLCnano system (Thermo Fisher Scientific) coupled to a Q-Exactive Plus mass spectrometer (Thermo Fisher Scientific) The peptides were separated by reversed-phase chromatography using a binary buffer system consisting of 0.1% FA (buffer A) and 90% ACN with 0.1% FA (buffer B). 1 μg of peptides were loaded on a 50 cm column with a 75 μM inner diameter and 2 μM C18 particles (EASY-spray PepMap, Thermo Fisher Scientific) and separated by a 170 min gradient (4-35% buffer B over 110 min, 35-90% buffer B over 1 min) at a flowrate of 300 nL/min. MS data were acquired using a data-dependent top-20 method with a maximum injection time (IT) of 50 ms, a scan range of 300–1650 m/z, and an AGC target of 3e6. The resolutions of the MS and MS/MS spectra were 70,000 and 17,500, respectively. The AGC for MS/MS acquisition was set at 5e4. The max IT and dynamic exclusion were set to 100 ms and 20 s, respectively.

Raw mass spectrometry data were processed with MaxQuant version v2.0.3.1 using the default setting if not stated otherwise (25). Oxidized methionine (M) and acetylation (protein N-term) were selected as variable modifications, and carbamidomethyl (C) as fixed modifications. Three missed cleavages for protein analysis were allowed. Label-free quantitation (LFQ) and “Match between runs” were enabled. Searches were performed against the mouse UniProt FASTA database (Martch 2021) containing 22,001 sequences. The proteinGroups output table was used for all proteomic analyses. Reverse proteins, proteins that were only identified by site, and potential contaminants were filtered out. The unique peptides were set at least ≥ 2. The protein groups were filtered to have at least 8 valid values (66.7%), the rest proteins that were identified in all 4 replicates of the Ctrl or LPS or LPS + Nbn were also retained. Eventually, a list of 2941 protein groups were used for all downstream analyses (**Table S1_Sheet** “After_filtering”). The Perseus software (26) was used to log2-transform the LFQ data, and the missing values were replaced using imputation based on the assumption of normal distribution with a downshift of 1.8 standard deviations and a width of 0.3 of the original ratio distribution (default setting) (**Table S1_Sheet** “Log2LFQ_annotation”). Proteins with a log2fold change ≥0.58 (upregulated) or ≤−0.58 (downregulated) and t test *p* value < 0.05 were selected as differentially expressed proteins (DEPs) (27, 28) (**Table S1_Sheet** “DEPs_LPSvsCtrl_Up”, “DEPs_LPSvsCtrl_Down”, “DEPs_LPS+NbnvsLPS_Up” and “DEPs_LPS+NbnvsLPS_Down”).

### Metabolomic analysis by GC-MS and LC-MS

For metabolomics analysis, dialyzed FBS (Gibco) was used instead of standard FBS. Cellular metabolites were extracted and analyzed according to previous established methods with modifications (24, 29). Briefly, iBMDMs were seeded at 1 × 10^6^ cells per well of a 6-well plate and allowed to attach for overnight. Cells were preincubated with 50 μM norbergenin for 1 h then treated with LPS (200 ng/mL) for 8 h. After treatment, cells were washed in cooled 0.9% NaCl and extracted in 1 mL 80% methanol with 0.3 nM pentaerythritol and 2.5 nM phenyl β-D-glucopyranoside as internal extraction standards. Extraction samples were incubated for 30 min at 4°C, then centrifuged for 10 min at 21,000 x g. The supernatants were dried in a SpeedVac system. The cell pellets were lysed by RIPA and used to measure protein levels for normalization purposes. 15 μL of methoxyamine hydrochloride solutions (40 mg dissolved in 1 mL pyridine) were added to the dried fraction and QC pools which were then incubated for 90 min at 30°C. Next, 60 μL of *N*-methyl-*N*-trimethylsilyltrifluoroacetamid (MSTFA) was added and incubated for 30 min at 37°C. Reaction mixtures were centrifuged for 10 min and 4°C at 21,000 x g and the supernatant was transferred to a glass vial with micro-insert. Measurement of metabolites was performed using established GC-MS standard protocol (30). The injection volume was 1 μL, and samples were injected at 1:5 split ratio. For a metabolite analysis, deconvolution of the total ion chromatogram, peak alignment and integration was performed using the software MS-DIAL v4.7 (31). Retention time index (RI) method in the peak identification was based on retention time of Alkanes mixture (C10-C40) as determined by MS-DIAL software. An in-house database with theoretical m/z and detected RI of known metabolites was used. RI tolerance was set to 20. We deducted the peaks of blank sample and all known artifact peaks (i.e., peaks caused by column bleed) in cell samples. Chromatographic peak area was normalized to internal standards and cell pellet concentration before multivariate analysis.

The analysis of cellular NAD^+^, NADH, NADP^+^, NADPH, GSH and GSSG was performed using a UltiMate 3000 UHPLC system coupled to an Orbitrap Elite mass spectrometer (LC-MS/MS, Thermo Fisher Scientific). iBMDMs were seeded at 1 × 10^6^ cells per well of a 6-well plate and allowed to attach for overnight. Cells were pretreated with norbergenin (50 μM) in the absence or presence of LPS (200 ng/mL). After another 8 h incubation, cells were extracted in 1 mL 80% methanol (-20°C) followed by centrifugation at 4°C for 10 min at 21,000 x g. The supernatant was dried in a SpeedVac system (Labogene). MS buffer (2% methanol and 0.1% FA) was added to the dried fraction and standards which were then centrifuged for 10 min at 21,000 x g and the supernatants were transferred to LC-MS vials. 5 μL of the sample was injected into a Accucore™ Vanquish C-18+ (100 × 2.1 mm, 1.5 µm particle size) UHPLC column, equipped with an Accucore™ Defender guards pk4 guard column (150 - C18 10 × 2.1 mm, 2.5 µm particle size (Thermo Fisher Scientific). The mobile phase system consisted of a mixture of solvent A (0.1% FA) and solvent B (LC-MS grade methanol). A gradient elution method was used for the analysis, 0–5 min 5% B, 5–65 min linear gradient to 100% B, keep 100% B for 39 min, and return to 5% B over 1 min. The flow was kept constant at 0.25 mL/min and the column was kept at 30°C throughout the analysis. MS analysis was performed in positive ion mode with the following parameters: Resolution, 60,000; spray voltage, 3.8 kV; capillary temperature, 300°C; sheath gas, 5; auxiliary gas, 2. The mass scanning range of the MS1 fullscan was set at 50–2000 m/z. The collision energy for collision-induced dissociation (CID) was set at 35 eV. Xcalibur software package (Thermo Fisher Scientific) and MS-DIAL were used to analyze the data.

### Bioinformatic analyses

The principal component analysis (PCA) plot, volcano plot and heatmap were performed using R. For the identification of enriched gene signatures, gene set enrichment analysis (GSEA) tool (Broad institute) was used. GSEA Preranked function was performed by comparison of normalized LFQ intensity data obtained from LPS + norbergenin vs LPS. Collections of signatures under the category H (hallmark gene sets) were screened and 1000 gene-set permutations for testing of significance was used. The enriched signaling pathways with adjusted *p*-value (FDR) < 0.05 were displayed. DAVID GO enrichment analysis was performed with DAVID v6.8 (https://david.ncifcrf.gov/tools.jsp) for the DEPs between LPS + Nbn vs LPS. The enrichment results (FDR < 0.05) can be found in **Table S1_Sheet** “GO_DEP_Up” and “GO_DEP_Down”. Downregulated DEPs of LPS+Nbn vs LPS were used for protein-protein interaction network, which was constructed utilizing the online STRING database (https://string-db.org). OPLS-DA and Pathway Analysis of metabolomics data were performed using the online tool MetaboAnalyst v5.0 (32), downregulated metabolites with VIP > 1.2 and FDR < 0.05 were then used for integrated analysis. The integrated proteomics and metabolomics analysis was performed through Joint-Pathway Analysis function within MetaboAnalyst, the enriched pathways (**Table S2**) with an impact > 0.4 and FDR < 0.05 were displayed.

### Statistical analysis

All experiments were performed in at least three biological independent replicates. All data are expressed as mean ± SEM. Statistical analyses were performed using Microsoft Excel, R or GraphPad Prism v9. The significant difference was calculated by the two-tailed unpaired student’s t-test or one-way ANOVA with Tukey’s HSD post-test. *p* ≤ 0.05 was considered statistically significant.

## Results

### Norbergenin suppressed cytokines and NO production in LPS-activated macrophages

**Figure 1:**
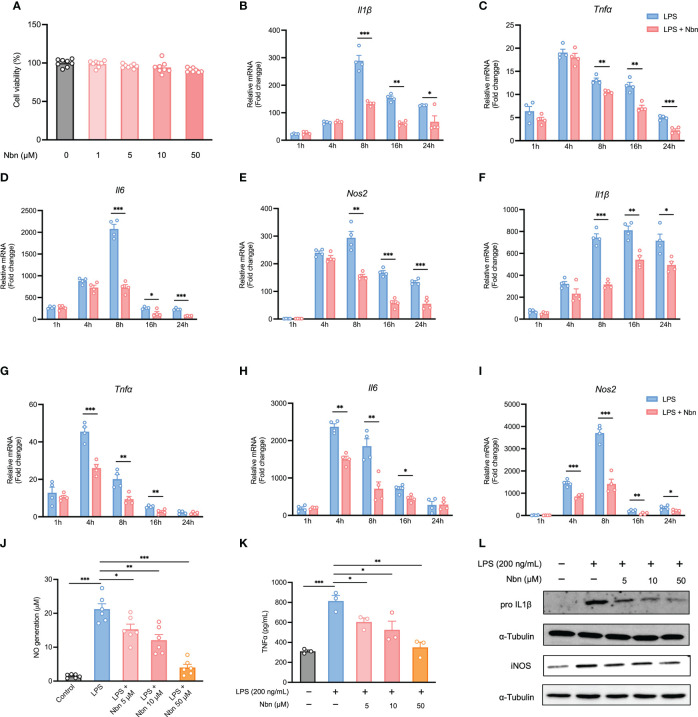
Norbergenin (Nbn) inhibits LPS-induced inflammation. **(A)**, Effect of Nbn at indicated concentrations on cell viability in iBMDMs. qPCR analysis of *Il1β*, *Tnfα*, *Il6*, and *Nos2* in iBMDMs **(B–E)** and BMDMs **(F–I)** pretreated with or without Nbn at concentrations of 50 μM for 1 h prior to stimulation with LPS (200 ng/mL) for indicated time points. NO generation **(J)**, TNFα generation **(K)**, and IL1β and iNOS **(L)** measured in iBMDMs preincubated with or without Nbn for 1 h followed by stimulation with LPS (200 ng/mL) by Griess assay, ELISA, and western blotting analysis, respectively. Data are presented as mean ± SEM of n ≥ 3 independent experiments. *p < 0.05, **p < 0.01, ***p < 0.001 (unpaired two-tailed student’s t test).

In order to select suitable concentrations of norbergenin for further experiments, we evaluated the cell viability of iBMDMs and BMDMs incubated with norbergenin at different concentrations (1, 5, 10 and 50 μM) for 24 h. As shown in **Figures 1A**, **S1A**, there were no significant toxic effects on iBMDMs and BMDMs at concentrations between 1 to 50 μM. Therefore, the concentrations 5, 10 and 50 μM of norbergenin were selected for the following experiments. LPS-elicited inflammatory cytokines and mediators directly drive inflammatory processes. To determine whether norbergenin has a role in preventing LPS-induced proinflammatory signaling in macrophages, we assessed its effects on LPS-induced inflammatory responses. The results showed that norbergenin in the presence of a concentration of 50 μM significantly inhibited the mRNA expression of *Il1β*, *Tnfα*, and *Il6* as well as TNFα cytokine production, the important inflammatory cytokine often overexpressed in response to LPS, both in iBMDMs (**Figures 1B–D, K**) and BMDMs (**Figures 1F–H**). In addition, a dose-dependent reduction of the expression of iNOS, an inflammatory enzyme regulating generation of NO, was observed in norbergenin treated iBMDMs (**Figure 1E**) as well as BMDMs (**Figure 1I**). In line with such changes, NO production (**Figure 1J**), the downstream biomarker of IL1β and iNOS protein expression (**Figures 1L**, **S1B**), were shown to be inhibited in norbergenin-treated inflammatory macrophages. Collectively, these results indicated that norbergenin could limit LPS stimulated inflammatory responses in both BMDMs and iBMDMs. We then decided to use iBMDMs for the further experiments to avoid the potential donor-to-donor biological variation associated with primary cells.

### Norbergenin altered proteomic profiles of inflammatory macrophages

**Figure 2:**
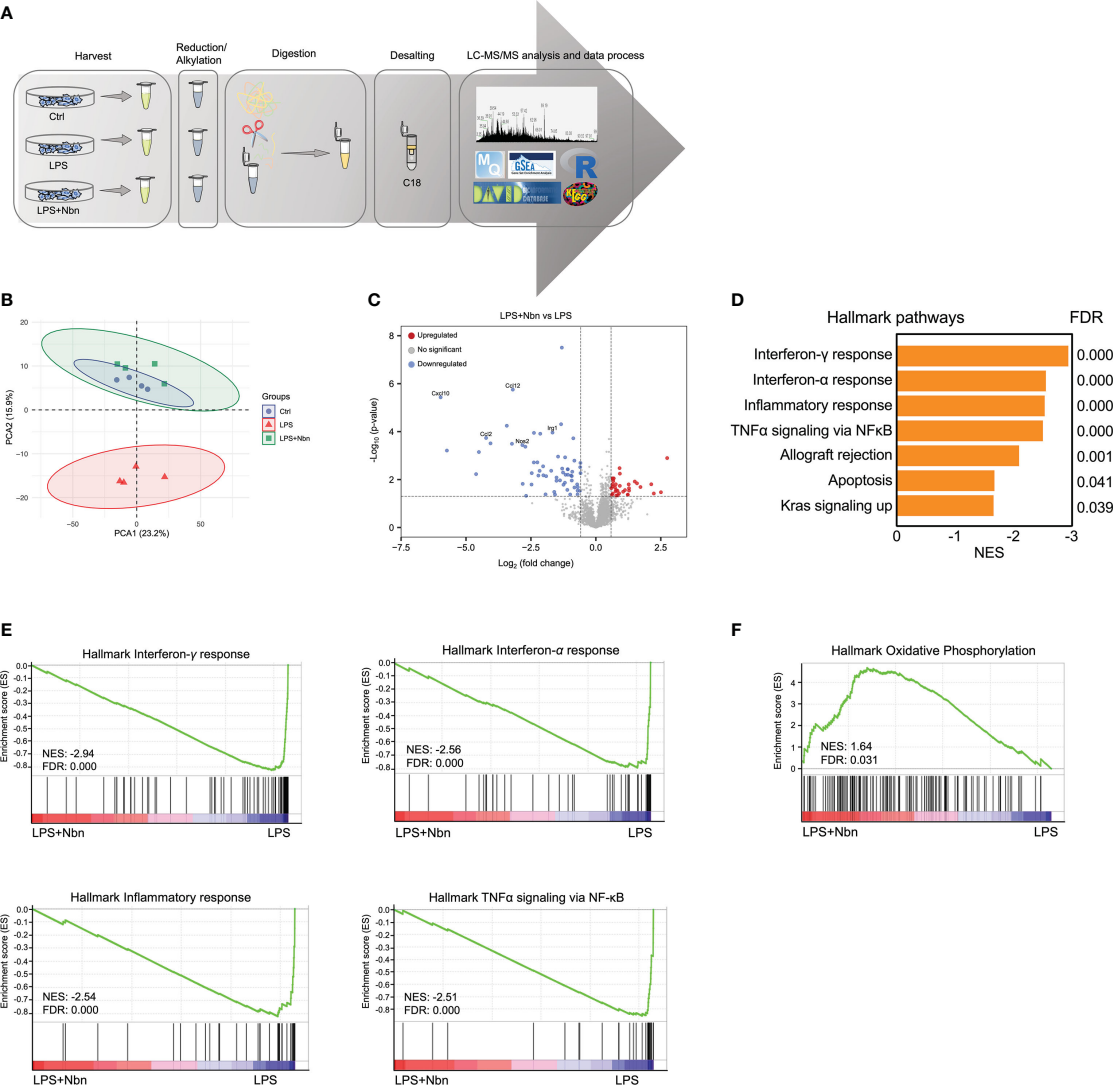
Norbergenin (Nbn) regulates global proteome associated with inflammatory process. **(A)**, Experimental design for proteomic analysis. **(B)**, PCA analysis of total identified proteins by proteomic analysis in iBMDMs pretreated with or without 50 μM Nbn followed by stimulation of LPS (200 ng/mL) for 8 h. **(C)**, Volcano plot showing proteins that were differentially expressed in iBMDMs pretreated with and without 50 μM Nbn for 1 h in the presence of LPS (200 ng/mL) for 8 h. Blue and red dots represent proteins significantly downregulated and upregulated (fold change > 1.5, *p* value < 0.05), respectively. **(D)**, GSEA of hallmark gene set from Molecular Signatures Database of the Broad Institute, showing the most enriched downregulated protein sets in LPS pretreated with norbergenin relative to only LPS-stimulated iBMDMs. Normalized enrichment scores (NES) are presented for pathways with false discovery rates (FDR) < 0.05. **(E)**, GSEA plots of interferon-γ response, interferon-α response, inflammatory response and TNFα signaling *via* NFκB protein signatures in Nbn-pretreated then LPS-stimulated iBMDMs relative to only LPS-stimulated iBMDMs from the analysis in **(D)** with members of the protein set presented in the ranked list of proteins. **(F)**, GSEA analysis of the “Oxidative phosphorylation” protein signatures. The x-axis of the plots**(E, F)** represents the ranking metric of the protein set, which is based on the correlation between the protein expression values and two different treatments. The y-axis shows the running enrichment score, which measures the degree to which the gene set is overrepresented at the top **(F)** or bottom **(E)** of the ranked protein list. Data are presented as mean ± SEM of n = 4 independent experiments.

To comprehensively understand the impacts of norbergenin on LPS-induced inflammatory macrophages and study the underlying mechanisms, we performed label-free proteomic analysis to characterize the altered proteins in control (Ctrl), LPS-induced macrophages pretreated with norbergenin (LPS + Nbn), or LPS alone (LPS) (**Figure 2A**). Based on the quantitative levels of 2,941 proteins after data filtering (**Table S1**, **Materials and Methods**), we observed a clear boundary between LPS and Ctrl or LPS + Nbn as shown by principal component analysis (PCA), primarily along PC2 (15.9%) (**Figure 2B**). Notably, there was no apparent separation between the Ctrl group and the LPS + Nbn group (**Figure 2B**), indicating that norbergenin-pretreated macrophages resembled the unstimulated control cells. We next identified 75 proteins that were upregulated in LPS-induced macrophages, whereas 16 were downregulated in these cells compared with controls (**Figure S2A**). Meanwhile, we found that 34 proteins were upregulated and 61 were downregulated in LPS + Nbn relative to LPS (**Figure S2A**). Of note, inflammatory-relevant proteins (CCL2, CCL4, CCL12, CXCL10 and NOS2) and itaconate-regulated protein immune-responsive gene 1 (IRG1) were among the most highly upregulated proteins induced by LPS (**Figure S2B**), whereas norbergenin pretreatment significantly reduced most of their expression levels (**Figure 2C**). To obtain insight into the overall protein functions modulated by norbergenin in response to LPS, 95 differentially expressed proteins (DEPs) were subjected to DAVID gene ontology (GO) analysis. GO enrichment analysis was performed mainly through biological process, cell composition and molecular function to annotate and enrich the downregulated or upregulated proteins. As shown in **Figure S2C**, the downregulated proteins have biological processes such as cellular response to interferon-beta, response to bacterium and immune system process, suggesting that norbergenin pretreatment attenuated the inflammatory responses stimulated by LPS. The cellular components were mainly involved in cytoplasm and nucleus, and the molecular functions were involved in ubiquitin protein ligase binding, CCR2 chemokine receptor binding and chemokine activity (**Figure S2C**). Upregulated proteins were highly enriched in biological processes related to DNA replication and cellular response to DNA damage stimulus (**Figure S2C**). The molecular functions were DNA-methyltransferase activity, DNA binding and chromatin binding (**Figure S2C**). Concerning cellular components, the identified proteins were mainly located in nucleoplasm (**Figure S2C**). These data suggested that norbergenin pretreatment diminished inflammatory responses and restored damaged cell replication induced by LPS.

We next explored the whole proteome *via* Gene Set Enrichment Analysis (GSEA) to identify the molecular pathways modulated by norbergenin in response to LPS. GSEA revealed that protein signatures in interferon-γ response, interferon-α response, inflammatory response and TNFα signaling *via* NF-κB were positively enriched in LPS-treated macrophages and associated proteins were downregulated in macrophages pretreated with norbergenin (**Figures 2D, E** and **Table 1**). Specifically, we observed that OXPHOS was identified as one of the top-ranked upregulated canonical pathways, and most relative proteins belonging to OXPHOS were augmented in norbergenin pretreated macrophages (**Figures 2F**, **S2D**). Initiation of inflammation in response to LPS requires adaption of cellular metabolism. This process involves multiple metabolic enzymes which catalyze metabolic reactions playing a causal role in inflammation progression (11). Typically activated macrophages exhibited increased glycolysis and a broken mitochondria associated with the reduced OXPHOS after LPS stimulation. In addition, IRG1 was observed to be upregulated in response to LPS, whereas norbergenin pretreatment strongly reduced its expression (**Figure S2E**). IRG1 codes for the enzyme cis-aconitate decarboxylase that converts aconitate (derived from citrate) to itaconate, which is known to impair sustained production of IL1β at later time points through a negative feedback mechanism and usually accumulated in LPS-stimulated macrophages (33). In addition, isocitrate dehydrogenase (IDH1) were observed to be downregulated in response to LPS (**Figure S2F**). Suppressed IDH representing a broken TCA cycle led to production of pro-inflammatory mediators (34). Norbergenin pretreatment slightly rescued the effect on this enzyme as shown by **Figure S2F**. Taken together, our proteomics data revealed that norbergenin pretreatment reduced inflammatory signaling pathways triggered by LPS and restored disordered mitochondria OXPHOS and metabolic enzymes.

**Table 1:** Selected DEPs in LPS+Nbn relative to LPS involved in inflammatory response, TNFα-NFκB pathway and metabolic pathway.

Category	Gene name	UniProt ID	Protein name	Log2 FC	Regulation
Inflammatory response	Znfx1	Q8R151	NFX1-type zinc finger-containing protein 1	-0.988	Down
	Epsti1	Q8VDI1	Epithelial-stromal interaction protein 1	-1.246	Down
	Lcp2	Q60787	Lymphocyte cytosolic protein 2	-1.192	Down
	Tnfaip2	Q61333	Tumor necrosis factor alpha-induced protein 2	-1.515	Down
	Isg20	Q9JL16	Interferon-stimulated gene 20 kDa protein	-1.380	Down
	Pml	Q60953	Protein PML	-1.514	Down
	Ifih1	Q8R5F7	Interferon-induced helicase C domain-containing protein 1	-1.914	Down
	Ube2L6	Q9QZU9	Ubiquitin/ISG15-conjugating enzyme E2 L6	-0.956	Down
	Rsad2	Q8CBB9	Radical S-adenosyl methionine domain-containing protein 2	-4.611	Down
	Ptgs2	Q05769	Prostaglandin G/H synthase 2	-2.469	Down
	Cdkn1A	P39689	Cyclin-dependent kinase inhibitor 1	-0.908	Down
	Trafd1	Q3UDK1	TRAF-type zinc finger domain-containing protein 1	-2.165	Down
	Icam1	P13597	Intercellular adhesion molecule 1	-1.082	Down
	Sp110	Q8BVK9	Sp110 nuclear body protein	-0.603	Down
	Peli1	Q8C669	E3 ubiquitin-protein ligase pellino homolog 1	-2.031	Down
	Parp14	Q2EMV9	Poly [ADP-ribose] polymerase 14	-0.827	Down
	Cd40	P27512	Tumor necrosis factor receptor superfamily member 5	-1.456	Down
	Ccl7	Q03366	C-C motif chemokine 7	-2.714	Down
	Helz2	E9QAM5	Helicase with zinc finger domain 2	-0.868	Down
	Cmpk2	Q3U5Q7	UMP-CMP kinase 2, mitochondrial	-2.141	Down
	Isg15	Q64339	Ubiquitin-like protein ISG15	-1.339	Down
	Cxcl10	P17515	C-X-C motif chemokine 10	-5.978	Down
	Ccl2	P10148	C-C motif chemokine 2	-4.226	Down
TNFα-NFκB pathway	Junb	P09450	Transcription factor jun-B	-1.937	Down
	Plek	Q9JHK5	Pleckstrin	-0.696	Down
	Tnfaip2	Q61333	Tumor necrosis factor alpha-induced protein 2	-1.515	Down
	Ifih1	Q8R5F7	Interferon-induced helicase C domain-containing protein 1	-1.914	Down
	Ptgs2	Q05769	Prostaglandin G/H synthase 2	-2.469	Down
	Cebpd	Q00322	CCAAT/enhancer-binding protein delta	-1.263	Down
	Cdkn1A	P39689	Cyclin-dependent kinase inhibitor 1	-0.908	Down
	Icam1	P13597	Intercellular adhesion molecule 1	-1.082	Down
	Cebpb	P28033	CCAAT/enhancer-binding protein beta	-1.082	Down
	Cxcl10	P17515	C-X-C motif chemokine 10	-5.978	Down
	Ccl2	P10148	C-C motif chemokine 2	-4.226	Down
	Sqstm1	Q64337	Sequestosome-1	-1.309	Down
Metabolic pathway	Cmpk2	Q3U5Q7	UMP-CMP kinase 2, mitochondrial	-2.141	Down
	Dck	P43346	Deoxycytidine kinase	-0.704	Down
	Hmox1	P14901	Heme oxygenase 1	-0.404	Down
	Irg1	P54987	Cis-aconitate decarboxylase	-1.669	Down
	Nos2	P29477	Nitric oxide synthase, inducible	-2.830	Down
	Ppat	Q8CIH9	Amidophosphoribosyltransferase	2.506	Up
	Cpt2	P52825	Carnitine O-palmitoyltransferase 2, mitochondrial	2.134	Up
	Dnmt3A	O88508	DNA (cytosine-5)-methyltransferase 3A	1.718	Up
	Cad	B2RQC6	Glutamine-dependent carbamoyl-phosphate synthase	0.895	Up
	Ndufa9	Q9DC69	NADH dehydrogenase 1 alpha subcomplex subunit 9	0.767	Up
	Polr2B	Q8CFI7	DNA-directed RNA polymerase II subunit RPB2	0.652	Up

### Norbergenin diminished LPS-induced proinflammatory signaling through downregulating NFκB and STAT3 signaling

**Figure 3:**
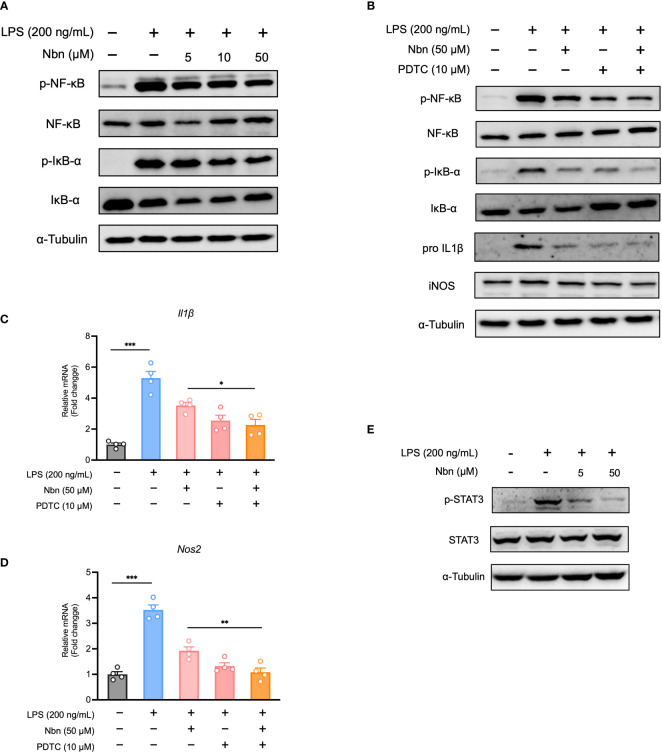
Norbergenin (Nbn) attenuates LPS-activated inflammation through inhibition of NFκB and STAT3 activation. **(A)**, Representative immunoblots of p-NFκB, NFκB, p-IκBα, IκBα measured in iBMDMs pretreated with or without Nbn for 1 h prior to incubation with LPS (200 ng/mL) for 4 h. **(B)**, iBMDMs were pretreated with or without PDTC for 30 min in the absence or presence of 50 μM Nbn before addition of LPS (200 ng/mL), p-NFκB, NFκB, p-IκBα and IκBα were analyzed by western blotting, IL1β and iNOS were measured by western blotting and qPCR **(B–D)** analysis. **E**, Representative immunoblots of p-STAT3 and STAT3. Data are presented as mean ± SEM of n ≥ 3 independent experiments. *p < 0.05, **p < 0.01, ***p < 0.001 (unpaired two-tailed student’s t test).

Prompted by GSEA results obtained from proteomics data, we determined whether NFκB was involved in anti-inflammatory effect of norbergenin. We found that several key proteins involved in NFκB pathway were among the downregulated DEPs from LPS + Nbn relative to LPS (**Table 1**). Furthermore, the increased expression levels of p-NFκB and p-IκBα by LPS were reduced by norbergenin in macrophages dose dependently (**Figures 3A**, **S3A**). In general, the phosphorylation of NFκB and IκBα are indicative of active inflammation. We then determined the extent to which the inhibition of NFκB activation may contribute to the preventive effects of norbergenin on inflammatory responses. For this aim, the NFκB specific inhibitor PDTC was used to suppress NFκB activation. The results revealed that PDTC diminished pro-inflammatory signaling on its own and could blunt impairment by norbergenin on LPS-induced the increased expression of p-NFκB and p-IκBα (**Figures 3B**, **S3B**). Moreover, PDTC treatment obviously increased the impacts of norbergenin on IL1β and iNOS both protein and mRNA expression levels induced by LPS in iBMDMs (**Figures 3B–D**, **S3C**). STAT3 has been reported to regulate cytokine-dependent inflammation *via* cross-talk with NFκB (9), we next investigated whether STAT3 was also involved in anti-inflammatory effects of norbergenin. Indeed, pretreatment of norbergenin was effective in decreasing STAT3 phosphorylation in iBMDMs stimulated with LPS (**Figures 3E**, **S3D**).

### Norbergenin inhibited MAPKs pathway in response to LPS

**Figure 4:**
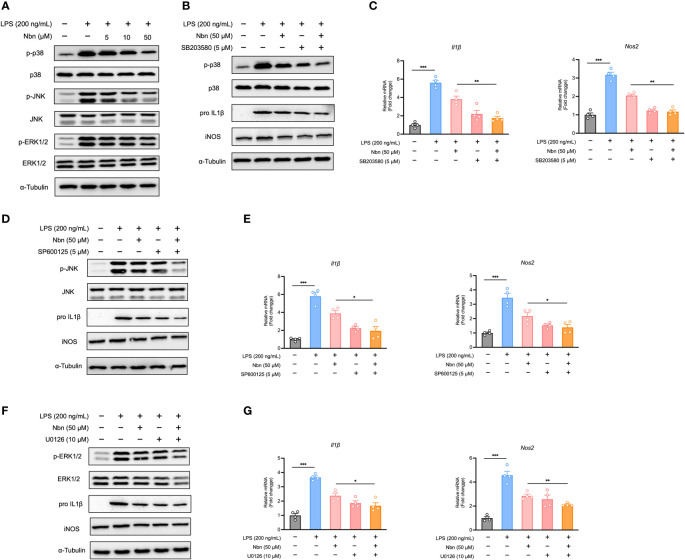
Norbergenin (Nbn) reduces LPS-triggered inflammation *via* inhibition of MAPKs in iBMDMs. iBMDMs were pretreated with or without Nbn at indicated concentrations for 1 h followed by treatment with LPS (200 ng/mL) for 8 h. **(A)**, Expressions of p-p38, p-JNK and p-ERK protein were determined by Western blotting, and p38, JNK and ERK were used as loading controls. iBMDMs were preincubated with or without Nbn (50 μM) for 1 h in the absence or presence of SB203580, SP600125, and U0126 followed by stimulation with LPS (200 ng/mL) for 8 h. Then the protein expression of p-p38 **(B)**, p-JNK **(D)**, and p-ERK **(F)** protein were determined by Western blotting, and p38, JNK, and ERK were used as loading controls. IL1β and iNOS protein or mRNA expression was assessed by western blotting or qPCR **(B–G)**. Data are presented as mean ± SEM of n ≥ 3 independent experiments. *p < 0.05, **p < 0.01, ***p < 0.001 (unpaired two-tailed student’s t test).

As MAPKs have also been shown to be a major regulator of inflammation (35), we reasoned that prevention of MAPK activation is also causative for the anti-inflammatory potential of norbergenin. Therefore, we measured the expression of proteins in the MAPK signaling pathways. As shown in **Figures 4A**, **S4A**, while LPS induced increases in expression of p-ERK, p-JNK, and p-p38 MAPK, norbergenin downregulated these proteins expression levels in a dose-dependent manner. We further measured the possible extent to which this could contribute to norbergenin-mediated suppression of pro-inflammatory markers. Blocking p38 MAPK, JNK, and ERK activation by using their inhibitors SB203580, SP600125, and U0126, respectively, potentiated the ability of norbergenin to decrease IL1β and iNOS protein expression levels as well as mRNA levels in LPS-activated iBMDMs (**Figures 4B–G**, **S4B–D**).

Overall, these data suggested that norbergenin alleviated inflammatory macrophage activation by limiting NFκB, STAT3, and MAPKs signaling.

### Norbergenin decreased the expression of TLR2 activated by LPS

**Figure 5:**
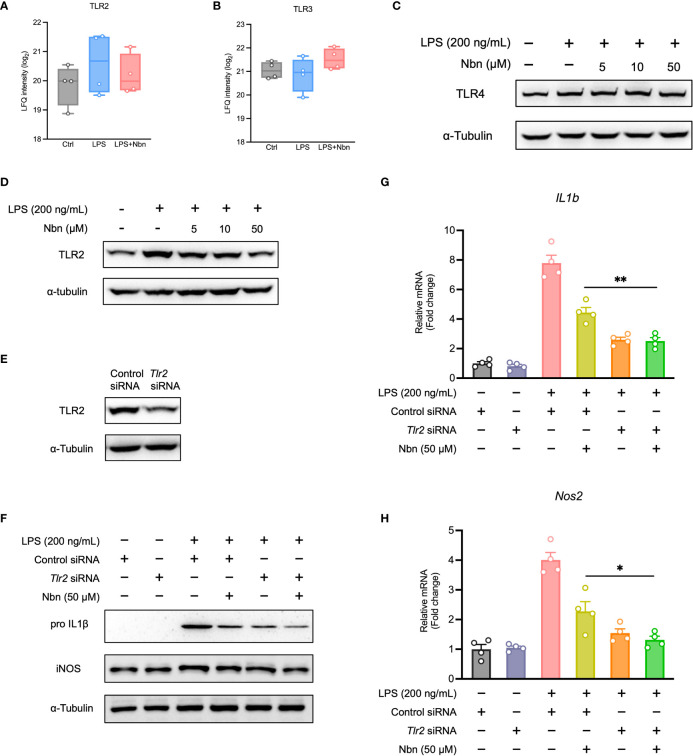
TLR2 involves anti-inflammatory response of LPS-activated macrophages to norbergenin (Nbn). Proteomic analysis of TLR2 **(A)** and TLR3 **(B)**. Representative immunoblots of TLR4 **(C)** and TLR2 **(D)** in iBMDMs pretreated with or without Nbn at indicated concentrations for 1 h then incubated with LPS (200 ng/mL) for another 8 h. RAW264.7 macrophages were transfected with control siRNA (30 nM) or TLR2 siRNA (30 nM) for 48 h prior to incubation with or without Nbn (50 μM) for 1 h in the absence or presence of LPS (200 ng/mL) for 8 h. Expression of TLR2 **(E)**, IL1β, and iNOS protein or mRNA was measured by western blotting **(F)** or qPCR analysis **(G, H)**, respectively. Data are presented as mean ± SEM of n ≥ 3 independent experiments. *p < 0.05, **p < 0.01 (unpaired two-tailed student’s t test).

Next, we investigated the effects of norbergenin treatment on TLRs expression in LPS-activated iBMDMs. Our proteomics data identified and quantified TLR2 and TLR3. As shown in **Figures 5A, B**, the expression of TLR2, not TLR3 was increased by LPS stimulation. Norbergenin pretreatment resulted in a weak reduction in TLR2 but not TLR3 in LPS-stimulated cells. We also measured TLR4, which is a critical TLRs member involved in LPS-induced inflammatory response. As shown in **Figures 5C**, **S5A**, LPS significantly induced TLR4 expression while norbergenin has no impact on its expression. Then we decided to focus on TLR2. The expression of TLR2 protein was observed to be diminished by norbergenin in a dose dependent manner in LPS-treated macrophages (**Figures 5D**, **S5B**). To further validate the role of TLR2 involved in norbergenin against LPS-induced pro-inflammatory signaling in macrophages, TLR2 siRNA was used to silence *Tlr2* gene expression (**Figures 5E**, **S5C**). Compared to control siRNA-transfection, *Tlr2* siRNA-transfection reduced the expression of IL1β and iNOS protein levels as well as their mRNA levels triggered by LPS, highlighting the importance of TLR2 involving in LPS-mediated inflammatory responses. When norbergenin was added prior to stimulation with LPS in *Tlr2* siRNA-transfected macrophages, these pro-inflammatory factors remain unaffected (**Figures 5F–H**, **S5D**), implying that TLR2 is required for the preventive effects of norbergenin on LPS-elicited pro-inflammatory signaling.

### Norbergenin restrained LPS-induced glycolysis and preserved mitochondrial OXPHOS in macrophages

**Figure 6:**
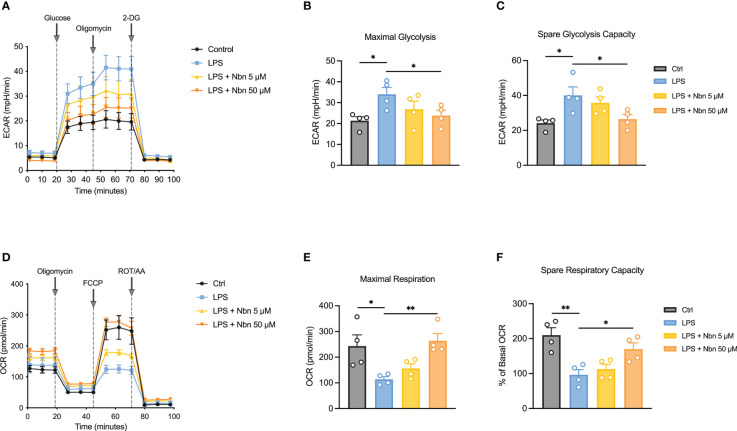
Norbergenin (Nbn) reverses LPS-induced disordered ECAR and OCR in iBMDMs. **(A)**, Representative ECAR in iBMDMs pretreated with or without Nbn (5 and 50 μM) followed by stimulation with LPS (200 ng/mL) for 8 h. Quantitative results of glycolysis **(B)** and glycolytic capacity **(C)**. **(D)**, Representative image of OCR in iBMDMs incubated with or without Nbn at concentrations of 5 and 50 μM for 1 h prior to stimulation with LPS (200 ng/mL) for another 8 h. Quantitative results of maximal respiration **(E)** and spare respiratory capacity **(F)**. Data are presented as mean ± SEM of n = 4 independent experiments. *p < 0.05, **p < 0.01 (unpaired two-tailed student’s t test).

LPS-induced macrophages adapt their metabolic pathways to drive the pro-inflammatory phenotype, such as enhanced glycolysis and reduced mitochondrial OXPHOS. Our proteomics data uncovered several downregulated DEPs involving inflammatory-related metabolic pathways (**Table 1**), such as itaconate biosynthesis (IRG1) and arginine biosynthesis (NOS2). The upregulated DEPs were mainly involved in purine and pyrimidine metabolism (PPAT, CAD and POLR2B), DNA methylation (DNMT3A), mitochondrial OXPHOS (NDUFA9) and fatty acid metabolism (CPT2), indicated that norbergenin pretreatment may restore the nucleotide synthesis and mitochondrial function interrupted by LPS. To further evaluate whether norbergenin could prohibit the disordered macrophage metabolism triggered by LPS, we measured the extracellular acidification rate (ECAR) and oxygen consumption rate (OCR) to directly estimate glycolytic metabolism and mitochondrial respiration. As seen in **Figures 6A–C**, norbergenin significantly diminished LPS-induced enhanced maximal glycolysis and spare glycolytic capacity in a dose-dependent manner in iBMDMs. As part of Warburg metabolism, a decreased OXPHOS is often observed along with elevation of glycolysis by LPS treatment. Our results indeed showed that LPS resulted in decreased OCR (**Figure 6D**), whereas norbergenin was found to be effective in increasing maximal respiration and spare respiratory capacity which were reduced by LPS stimulation in macrophages (**Figures 6E, F**). These data indicated that norbergenin attenuated glycolysis and preserved mitochondrial oxidation in response to LPS in macrophages.

### Norbergenin altered metabolic profiles of inflammatory macrophages

**Figure 7:**
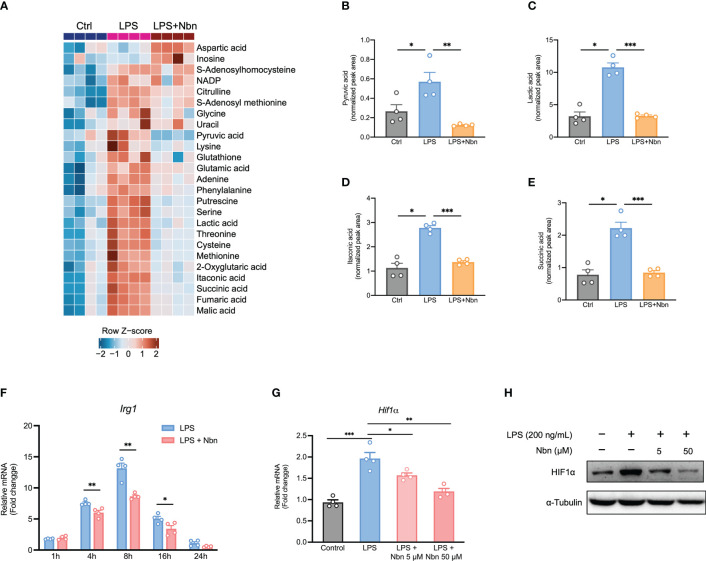
Norbergenin (Nbn) alters LPS-triggered metabolic profiles in iBMDMs. **(A)**, Heatmap of selected significant metabolites in control (Ctrl), LPS (200 ng/mL)-stimulated (8 h) and Nbn (50 μM)-pretreated (1 h) then LPS (200 ng/mL)-stimulated (8 h) iBMDMs. The rows represent different metabolites, and the log-transformed normalized metabolite intensities were scaled by z-scoring. **(B–E)** Corresponding metabolites from **(A)**. **(F)**, qPCR analysis of *Irg1* expression in iBMDMs pretreated with or without 50 μM Nbn followed by incubation of LPS (200 ng/mL) for indicated time points. **(G)**, HIF1α mRNA and protein **(H)** expression measured in iBMDMs pretreated with or without Nbn (5 and 50 μM) for 1h then stimulated with LPS (200 ng/mL) for 8 h, respectively. Data are presented as mean ± SEM of n = 4 independent experiments. *p < 0.05, **p < 0.01, ***p < 0.001 (unpaired two-tailed student’s t test).

Next, we performed metabolomic analysis to evaluate the metabolic profiles in these different treatment groups. Similar to proteomics data, the metabolic profiles in LPS-stimulated macrophages pretreated with and without norbergenin were obviously separated from each other (**Figure S6A**). Cells in the presence of norbergenin upon LPS stimulation were closest to the unstimulated naïve macrophages (**Figure S6A**). One-way ANOVA analysis revealed that there were 25 metabolites (FDR < 0.05) varying globally among these differentially treated macrophages (**Figure 7A**). The analysis also showed that norbergenin abolished cytosolic pyruvate and lactate buildups which are closely related to inflammatory activation (**Figures 7A–C**). In line with our proteomics data, itaconate production was accumulated by LPS stimulation, whereas norbergenin pretreatment notably blocked its production (**Figures 7A, D**). Consistent with the impact on itaconate production, norbergenin was found to be effective in reducing IRG1 expression both at protein and mRNA levels that were induced by LPS (**Figures 7F**, **S2E**). Succinate promotes HIF1α stabilization and sustains IL1β production, suggesting its instrumental role in macrophage activation. Norbergenin supplementation was found to be effective in dampening succinate abundance (**Figures 7A, E**). This result prompted us to study the impact of norbergenin on HIF1α activation. As expected, LPS-stimulation increased HIF1α mRNA and protein expression levels while norbergenin pretreatment decreased this upregulation at mRNA and protein levels (**Figures 7G, H**, **S3D**). Additionally, elevated arginine and citrulline levels were found in macrophages treated with LPS, but norbegenin treatment decreased these elevations of both metabolites (**Figures 7A**, **S6B, S6C**). Variable importance in projection (VIP) scores >1.2 indicated that levels of 16 differentially expressed metabolites (DEMs) including putrescine, itaconate and TCA cycle intermediates were the largest contributors to discrimination between LPS pretreated with norbergenin and LPS alone (**Table 2**). Furthermore, we performed pathway enrichment analysis on these significantly decreased metabolites by norbergenin pretreatment in macrophages upon LPS stimulation. The results indicated that highly enriched pathways were related to arginine biosynthesis, TCA cycle, alanine, aspartate and glutamate metabolism, pyruvate metabolism, and glycine and serine metabolism (**Figure S6D** and **Table S2**). Notably, some recent studies (36, 37) reported that serine metabolism supported LPS-induced IL1β generation, therefore, it is considered to be associated with inflammatory responses. Collectively, these findings revealed that norbergenin pretreatment could reverse metabolic remodeling triggered by LPS to exert its anti-inflammatory effects in macrophages.

**Table 2:** Differentially expressed metabolites (DEMs) from LPS-induced macrophages pretreated with and without norbergenin.

no.	Metabolite	Molecular formula	KEGG	VIP^a^	Log2(FC)	*P*value^b^	FDR^c^	Regulation
1	Putrescine	C4H12N2	C00134	1.455	-2.638	0.000	0.003	Down
2	Itaconic acid	C5H6O4	C00490	1.450	-1.197	0.000	0.000	Down
3	Malate	C4H6O5	C00149	1.428	-0.893	0.000	0.003	Down
4	Serine	C3H7NO3	C00716	1.425	-1.375	0.001	0.003	Down
5	Cysteine	C3H7NO2S	C00097	1.417	-2.224	0.003	0.012	Down
6	Succinic acid	C4H6O4	C00042	1.411	-1.308	0.001	0.005	Down
7	Threonine	C4H9NO3	C00188	1.410	-1.093	0.001	0.003	Down
8	Fumaric acid	C4H4O4	C00122	1.409	-1.035	0.000	0.003	Down
9	Lactic acid	C3H6O3	C00186	1.394	-1.520	0.000	0.003	Down
10	Pyruvic acid	C3H4O3	C00022	1.360	-2.134	0.008	0.026	Down
11	2-Oxyglutaric acid	C5H8O5	C00026	1.331	-0.973	0.004	0.013	Down
12	Arginine	C6H14N4O2	C00062	1.330	-0.590	0.003	0.012	Down
13	Citrulline	C6H13N3O3	C00327	1.317	-0.937	0.001	0.004	Down
14	Glutathione	C10H17N3O6S	C00051	1.210	-0.873	0.009	0.027	Down
15	Inosine	C10H12N4O5	C00294	1.222	0.943	0.030	0.078	Up
16	Methionine	C5H11NO2S	C00073	1.314	-1.532	0.032	0.078	Down

a The VIP value was obtained from OPLS-DA model with a threshold of 1.2.

b *P*value from t-test.

c FDR was obtained from the adjusted T-test P value of FDR correction by Benjamini-Hochberg method.

**Figure 8:**
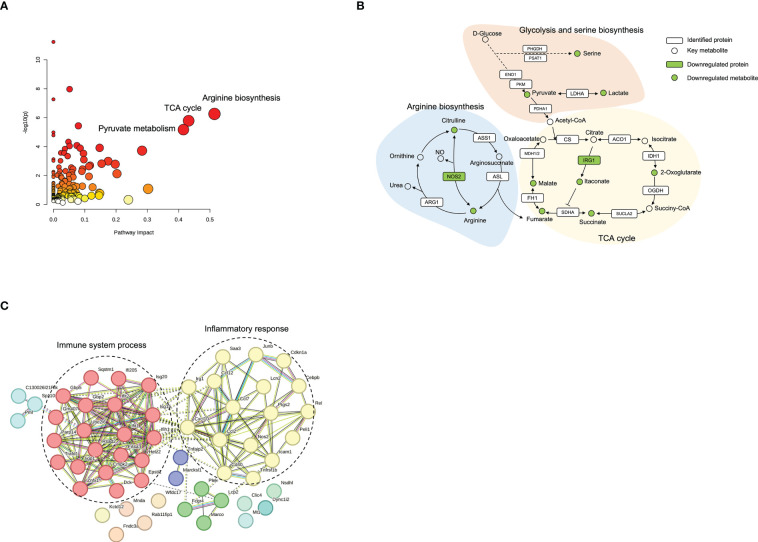
Integrated analysis of proteomics and metabolomics datasets. **(A)** Enriched pathways from Joint-pathway analysis. **(B)** The perturbed proteins and metabolites corresponding metabolic pathways related to norbergenin pretreatment. **(C)** Protein-protein interaction network analysis of 61 downregulated DEPs involved in LPS+norbergenin and LPS groups.

To provide a comprehensive understanding of biological systems in response to norbergenin pretreatment, proteomics and metabolomics data were integrated to acquire the shared pathways and interaction between downregulated DEPs and downregulated DEMs. The systematic scheme of regulated metabolic pathways and related proteins under norbergenin pretreatment were summarized (**Figures 8A, B** and **Table S2**). The results indicated that the disorder of arginine biosynthesis, TCA cycle and pyruvate metabolism were involved in anti-inflammatory effects of norbergenin. Several key metabolites, including pyruvate, lactate, TCA cycle intermediates, arginine and citrulline, were identified among the downregulated DEMs (**Figure 8B**). In addition, two DEPs (NOS2 and IRG1) that were found to be involved in these metabolic pathways (**Figure 8B**). Instead, most of DEPs were discovered in inflammatory response and immune system process (**Figure 8C**). Taken together, our proteomics and metabolomics data respectively interpreted the anti-inflammatory effects of norbergenin from the perspective of canonical inflammatory signaling cascades and metabolic reprogramming.

## Discussion

Inflammation is a common feature of many chronic disorders, such as obesity, insulin resistance, atherosclerosis and cancer. There is strong evidence that utilization of natural products could ameliorate chronic inflammation (38, 39). In this respect, we found that norbergenin opposed LPS-induced macrophage activation by suppressing NFκB, MAPKs, and STAT3 activation. Importantly, inhibition of metabolism in activated macrophages is known to offer protection against inflammation (40, 41). Likewise, here we also report that treatment with norbergenin prevented cellular metabolic remodeling in response to LPS.

As a widespread natural product, norbergenin has been proven to possess multiple biological functions, especially the capability of scavenging free radicals activity may contribute directly or indirectly to its reported anti-inflammatory effect (42). Inflammatory macrophages exposed to LPS are well characterized by producing a wide range of pro-inflammatory cytokines and mediators which are direct drivers for inflammatory state (43, 44). Norbergenin was observed to inhibit the production of pro-inflammatory cytokines, such as IL1β, IL6, TNFα, and mediator NO in iBMDMs induced by LPS. Similar effects on pro-inflammatory cytokines were also observed in BMDMs. Furthermore, our proteomic analysis also revealed that norbergenin downregulated LPS-induced inflammation-associated proteins levels. Interestingly, norbergenin was shown to revert the changes in cellular metabolism induced by LPS stimulation in macrophages. This becomes even more important with respect to the critical role of metabolic reprogramming for macrophage effector functions. It is well established that cellular metabolic changes are required for inflammatory macrophage activation by LPS (45, 46). Some studies have recently demonstrated that altering metabolic reprogramming could be beneficial for the development of inflammation (1, 14). Macrophages activated by LPS generally rely on aerobic glycolysis to generate energy, in contrast to resting macrophages which instead rely on mitochondrial OXPHOS. Norbergenin treatment reduced LPS-elicited high glycolysis and facilitated lowered OXPHOS in iBMDMs, which is consistent with our proteomics and metabolomics data. Collectively, these data demonstrated that norbergenin could alleviate proinflammatory signaling by limiting pro-inflammatory factors and modulating metabolic remodeling caused by LPS stimulation in macrophages.

It should be mentioned that metabolic reprogramming induced by LPS activation in macrophages is usually associated with accumulation of specific metabolites (47). These metabolites, produced by their respective enzymes, themselves act as signaling molecules which in turn affect inflammatory processes (40). For example, citrate resulting from the first break of TCA cycle at IDH1 is shifted toward itaconate generation, and itaconate produced by IRG1 is thought to be an anti-inflammatory mediator due to its activation of anti-inflammatory transcription factor NRF2 to limit inflammatory cytokines (11, 33, 48). Itaconate also is reported in the context of inhibition of succinate dehydrogenase (SDH), a crucial pro-inflammatory regulator (15, 49). As expected, metabolic analysis showed that itaconate accumulated at high levels within macrophages stimulated by LPS. In support of these altered itaconate levels, higher expression of IRG1 was correspondingly detected by proteomic analysis and qPCR in LPS-challenged macrophages. In contrast, the effects of LPS on itaconate level and IRG1 activation were alleviated when cells were incubated with norbergenin. The second break of TCA cycle induced by suppression of SDH leads to succinate accumulation. Succinate drives inflammatory signaling by stabilizing HIF1α and consequently upregulating IL1β production (14, 50). In line with our results, LPS elicited significant increases in the level of succinate. Such change could be rescued in macrophages treated with norbergenin. The reduction of succinate levels could help to explain the similar effects observed in its downstream protein HIF1α and IL1β expression levels which were markedly decreased by norbergenein pretreatment. In contrast to these metabolites, activated macrophages exhibited high pyruvate and lactate levels which means improved glycolysis in LPS-induced macrophages (13, 51). Indeed, we found that macrophages with LPS treatment displayed accumulation of lactate and pyruvate abundance. However, norbergenin totally abolished the impact of LPS on glycolysis and reduced related metabolites pyruvate and lactate accumulation, allowing to maintain proper glycolysis in LPS-treated macrophages. This is also reflected on the impact of norbergenin on ECAR in LPS-stimulated macrophages. Accompanying enhanced aerobic glycolysis, mitochondrial oxidation is impaired in activated macrophages, norbergenin could also reverse this influence of LPS on mitochondrial OXPHOS.

It is well known that arginine is converted into NO and citrulline by iNOS, with citrulline being directed into the citrulline-NO cycle for NO generation in inflammatory macrophages (52, 53). These results were further confirmed by the observations that macrophages receiving LPS treatment showed significantly higher citrulline and arginine levels as well as higher *Nos2* expression levels compared with that of control cells. Here, our analysis showed that administration of norbergenin decreased the levels of citrulline and arginine induced by LPS in macrophages. Accordingly, expression of iNOS and NO production were also presented to be decreased by norbergenin in LPS-activated macrophages. In particular, serine metabolism was demonstrated to be linked to LPS-stimulated inflammation. This effect attributes to the generation of glycine from serine which promotes glutathione-synthesis to maintain cellular redox balance and supports cytokine IL1β production (36, 37). We found that norbergenin downregulated the activation of glycine and serine metabolism by LPS. Taken together, our data indicated that norbergenin could ameliorate proinflammatory signaling through opposing the metabolic changes elicited by LPS which support the involvement of pro-inflammatory cytokines in macrophages.

It is well established that macrophage activation by LPS is highly dependent on TLRs. TLRs are generally known as crucial components to detect pathogens and activate immune responses. Among these receptors, TLR2 and TLR4 are considered the most widely expressed members in TLR families. Engagement of TLR2 and TLR4 is found to induce NFκB and MAPKs pathway, promote gene expression and release of inflammatory-driving mediators release (5). Most recently, TLR2 has been shown to contribute to the regulation of metabolic reprogramming in inflammatory macrophages (54), likely providing a basis for subsequent inflammatory responses including altered signaling and metabolic pathways. Here, we showed that TLR2 and TLR4 expression levels increased in proteomic analysis or immunoblotting in LPS-treated macrophages. However, results indicate that TLR2, but not TLR4, is involved in preventive effect of norbergenin on LPS-stimulated proinflammatory signaling in macrophages. Further studies are planned to investigate the specificity of norbergenin in more detail. Within macrophages, NFκB serves as a master regulator of inflammatory process. Activated NFκB released from the phosphorylated and subsequently degraded IκBα protein is translocated into nucleus, where it binds to promoter regions of many genes, leading to activation of inflammatory genes, such as *Cox2*, *Nos2*, *Il1β*, *Il6*, etc. (35, 38). Unsurprisingly, proteins connected to NFκB pathway were highly enriched in our proteomics data showing its close relation to LPS-stimulated proinflammatory signaling. In line with these data, examination of protein expression revealed a marked increase in the extent of p-NFκB and p-IκBα in macrophages treated with LPS. By employing NFκB-specific inhibitor PDTC, we found that suppression of NFκB and IκBα enhanced the impact of norbergenin on IL1β, iNOS expression affected by LPS. In addition to induced NFκB, LPS is reported to initiate MAPK signaling cascades. Importantly, MAPKs regulate pro-inflammatory enzyme-, cytokine- and chemokine-production in inflammatory progression (55, 56). Here, we provided evidence for increased p-ERK, p-JNK and p-p38 protein expression levels increasing in macrophages in response to LPS, with a concomitant elevation of inflammatory cytokines. Norbergenin treatment resulted in impairment of these proteins activation and inhibition of IL1β and iNOS expression. Furthermore, their individual inhibitors of ERK, JNK and p38 substantially potentiated the capability of norbergenin to repress IL1β and iNOS expressions upon LPS exposure. Beyond the engagement of NFκB and MAPKs signaling in the anti-inflammatory effects of norbergenin, our results also supported STAT3 inhibition to be associated with this effect. It is noteworthy that STAT3 activation contributes to IL1β and IL6 production through inducing PKM2 (57, 58). We found that norbergenin decreased the activation of STAT3 in LPS-stimulated macrophages. This might partially explain the inhibitive effects of norbergenin on IL1β and IL6 production. Overall, our results suggested that norbergenin supplementation could impair NFκB and MAPKs signaling pathways and dampen STAT3 phosphorylation in macrophages, thus reducing LPS-induced proinflammatory signaling.

**Figure 9:**
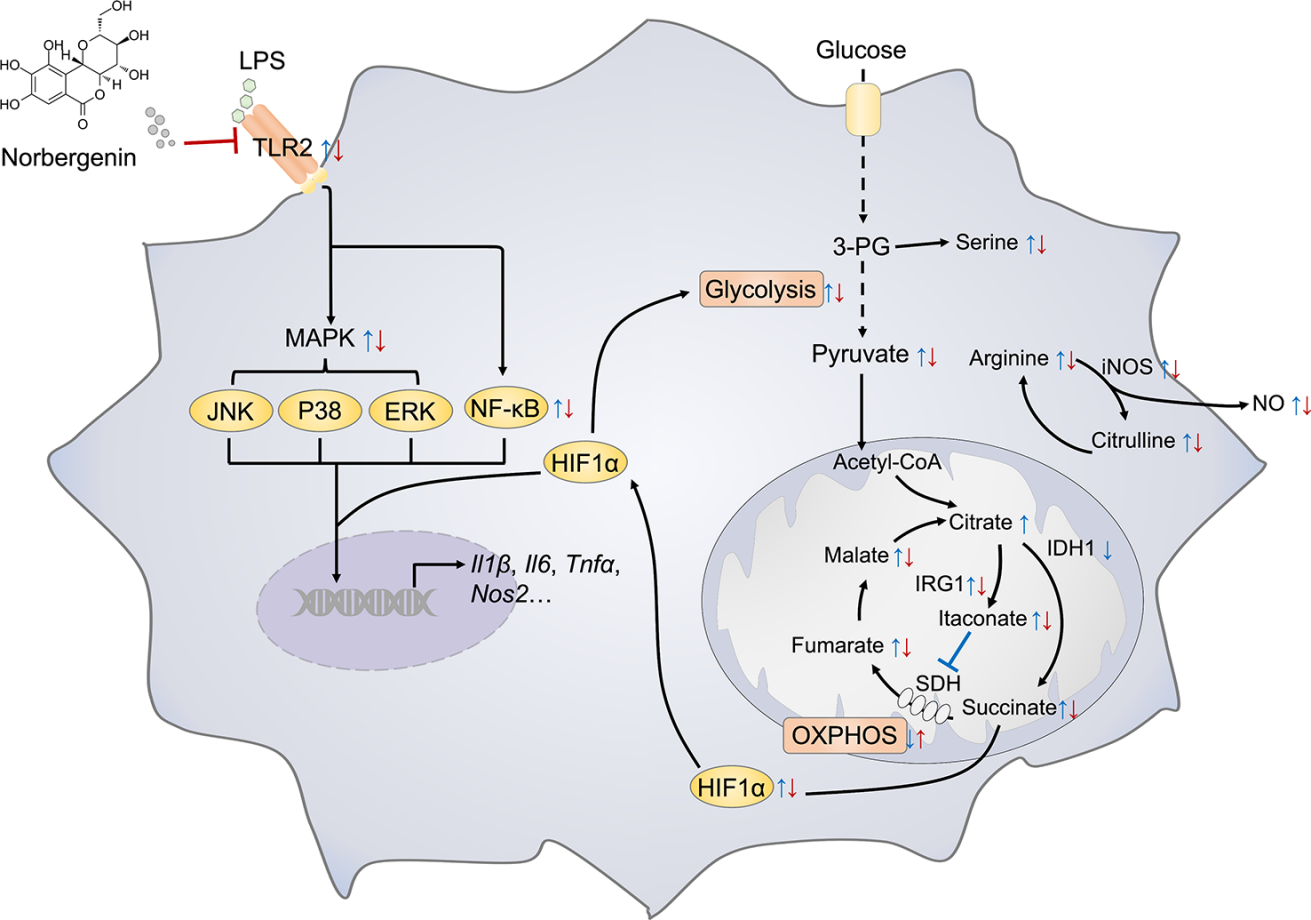
Schematic representation illustrating the mechanism of norbergenin (Nbn) preventing inflammation in macrophages. Key metabolite and inflammatory pathways that were significantly regulated by LPS or LPS + Nbn are shown. Blue arrow indicates the metabolite expression of LPS vs Control (Ctrl). Red arrow indicates the metabolite expression of LPS + Nbn vs LPS. The arrow direction indicates the increase (↑) or decrease (↓) of the corresponding metabolite caused by LPS vs Ctrl or LPS + Nbn vs LPS.

In summary, as depicted in **Figure 9**, we demonstrated that norbergenin limited LPS-caused inflammatory responses in macrophages by repression of TLR2-dependent NFκB, MAPKs and STAT3 signaling. Moreover, norbergenin reversed metabolic remodeling, restrained enhanced aerobic glycolysis, potentiated reduced OXPHOS, and restored TCA cycle. Furthermore, norbergenin regulated altering metabolites and metabolic enzymes responding to LPS in macrophages. Our work has uncovered functionally and mechanistically the effect of norbergenin on LPS-induced proinflammatory signaling and provides a novel perspective for metabolic intervention by natural products against inflammatory progress.

## Data availability statement

The datasets presented in this study can be found in online repositories. The mass spectrometry-based proteomics data have been deposited to the ProteomeXchange Consortium (http://www.proteomexchange.org/) with the dataset identifier PXD040202. Metabolomics data have been deposited to the EMBL-EBI MetaboLights database (https://www.ebi.ac.uk/metabolights) with the identifier MTBLS7127.

## Author contributions

WL, ZC, and WW conceived and designed the study; WL and ZC performed the experiments; FS isolated and provided norbergenin for preliminary experiments; FS, SB, and MB helped with instruments and techniques; EH provided necessary tools, supervised, and supported experiments; WL, ZC, and WW wrote the manuscript. All authors contributed to the article and approved the submitted version.
