# Supplementary material for: Norbergenin prevents LPS-induced inflammatory responses in macrophages through inhibiting NFκB, MAPK and STAT3 activation and blocking metabolic reprogramming
Source: Front Immunol. 2023 May 12;14:1117638. doi: 10.3389/fimmu.2023.1117638 (PMC10213229; doi:10.3389/fimmu.2023.1117638)

Norbergenin prevents LPS-induced inflammatory responses in macrophages through inhibiting NFκB, MAPK and STAT3 activation and blocking metabolic reprogramming

Wan Li^1,2^, Zhengnan Cai^1,2^, Florian Schindler^1,3^, Sheyda Bahiraii^3,4^, Martin Brenner^1,3,4^, Elke H. Heiss^4^, Wolfram Weckwerth^1,5,*^

^1^Molecular Systems Biology (MOSYS), Department of Functional and Evolutionary Ecology, University of Vienna, Vienna, Austria

^2^Vienna Doctoral School of Ecology and Evolution, University of Vienna, Vienna, Austria

^3^Vienna Doctoral School of Pharmaceutical, Nutritional and Sports Sciences, University of Vienna, Vienna, Austria

^4^Department of Pharmaceutical Sciences, University of Vienna, Vienna, Austria

^5^Vienna Metabolomics Center (VIME), University of Vienna, Djerassiplatz 1, 1030 Vienna, Vienna, Austria

*Address Correspondence to: Wolfram Weckwerth

(wolfram.weckwerth@univie.ac.at)

**Table S3 Primers used for real-time qPCR.**

| Gene | Forward 5' to 3' | Reverse 5' to 3' |
| --- | --- | --- |
| *Il1β* | TGGCAACTGTTCCTG | GGAAGCAGCCCTTCATCTTT |
| *Il6* | ACAAAGCCAGAGTCCTTCAGAGAG | TTGGATGGTCTTGGTCCTTAGCCA |
| *Tnfα* | TCTGTCTACTGAACTTCGGGGTGA | TTGTCTTTGAGATCCATGCCGTT |
| *Rps9* | GCAAGATGAAGCTGGATTAC | GGGATGTTCACCACCTG |
| *Nos2* | CAGAGGACCCAGAGACAAGC | TGCTGAAACATTTCCTGTGC |
| *Irg1*  *Hif1α* | GCAACATGATGCTCAAGTCTG  GTCCCAGCTACGAAGTTACAGC | TGCTCCTCCGAATGATACCA  CAGTGCAGGATACACAAGGTTT |

**Figure legends**

**Figure S1**. The effects of norbergenin (Nbn) on cell viability and protein expression in BMDMs or iBMDMs. **A**, The cell viability of BMDMs. **B**, Quantitative analysis of IL1β and iNOS protein levels in iBMDMs. Data are presented as mean ± SEM of n ≥ 3 independent experiments. **p* < 0.05, ***p* < 0.01, ****p* < 0.001 (unpaired two-tailed student’s t test).

**Figure S2.** Norbergenin (Nbn) regulates global proteome associated with inflammatory process. **A**, Quantitative results of identified proteins by proteomics analysis. **B**, Volcano plot showing proteins that were differentially expressed in LPS (200 ng/mL) treated iBMDMs and control (Ctrl). Blue and red dots represent proteins significantly downregulated and upregulated (fold change > 1.5, p value < 0.05), respectively. **C**, GO annotation analysis of DEPs in Nbn pretreated iBMDMs relative to LPS-stimulated iBMDMs alone. **D**, Heatmap of the representative proteins of illustrated GSEA terms from OXPHOS term. **E**, LFQ intensity of IRG1. **F**, LFQ intensity of IDH1. The LFQ intensities were log2 transformed. Data are presented as mean ± SEM of n = 4 independent experiments. ****p* < 0.001 (unpaired two-tailed student’s t test).

**Figure S3.** The effects of norbergenin (Nbn) on protein expression levels in NFκB pathway in LPS-treated iBMDMs. **A-C**, Quantitative analysis of protein expression levels in NFκB pathway in iBMDMs. **D**, Quantitative analysis of STAT3 and HIF1α protein expression. Data are presented as mean ± SEM of n ≥ 3 independent experiments. **p* < 0.05, ***p* < 0.01, ****p* < 0.001 (unpaired two-tailed student’s t test).

**Figure S4.** The effects of norbergenin (Nbn) on MAPK protein expression levels in LPS-treated iBMDMs. **A-D**, Quantitative analysis of MAPK protein levels in iBMDMs. Data are presented as mean ± SEM of n = 3 independent experiments. **p* < 0.05, ***p* < 0.01, ****p* < 0.001 (unpaired two-tailed student’s t test).

**Figure S5.** The effects of norbergenin (Nbn) on TLR2 signaling in LPS-induced iBMDMs. **A-D,** Quantitative analysis of TLR4, TLR2, IL1β and iNOS protein expression levels in iBMDMs. Data are presented as mean ± SEM of n = 3 independent experiments. **p* < 0.05, ***p* < 0.01, ****p* < 0.001 (unpaired two-tailed student’s t test).

**Figure S6**, Norbergenin (Nbn) alters LPS-triggered metabolic profiles in iBMDMs. **A**, PCA analysis of total identified metabolites among control (Ctrl), LPS and LPS pretreated with norbergenin (LPS + Nbn). Metabolite abundance of arginine (**B**) and citrulline (**C**). **D**, Pathway enrichment analysis of 16 significantly reduced metabolites (VIP > 1.2, FDR < 0.05) between LPS + Nbn and LPS, and top 25 clusters with their representative enriched terms are shown. Data are presented as mean ± SEM of n = 4 independent experiments. **p* < 0.05, ***p* < 0.01, ****p* < 0.001 (unpaired two-tailed student’s t test).

**Figure S1**


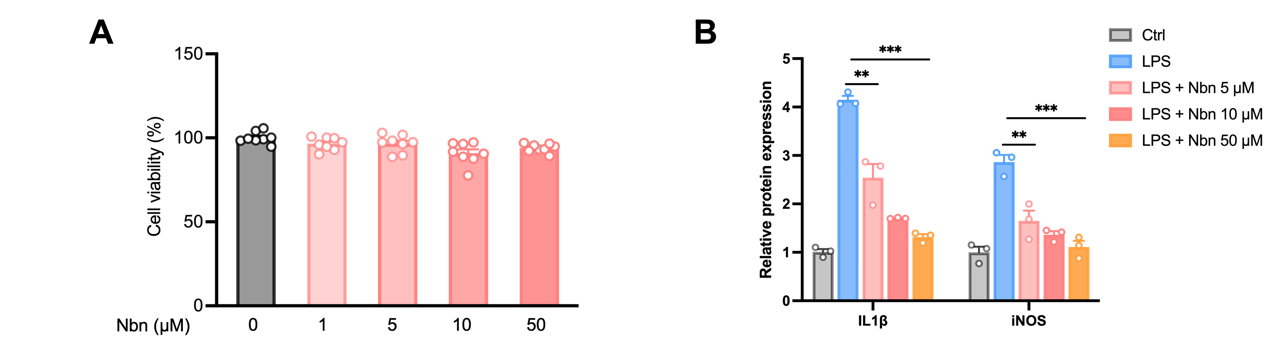


**Figure S2**


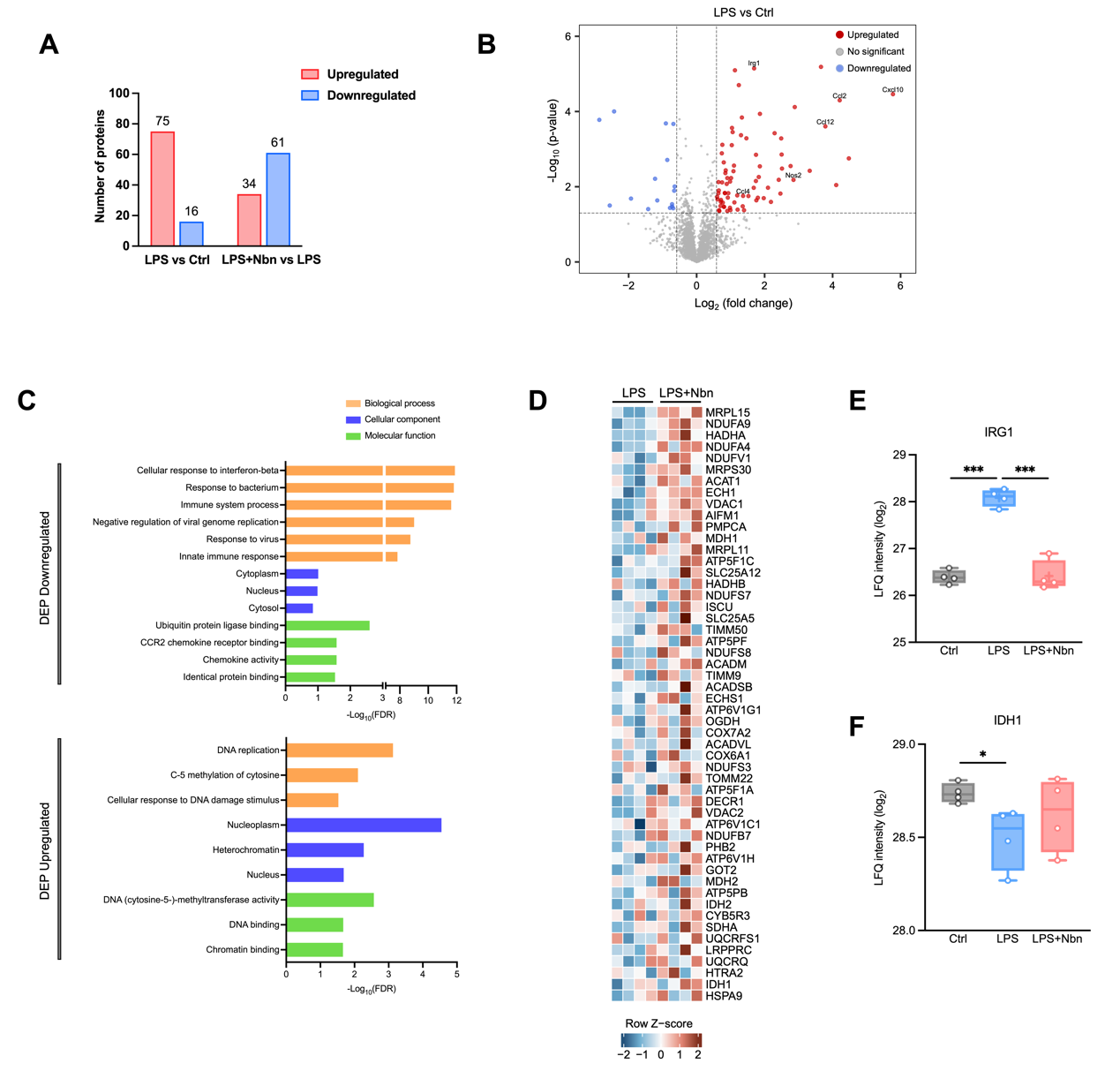


**Figure S3**


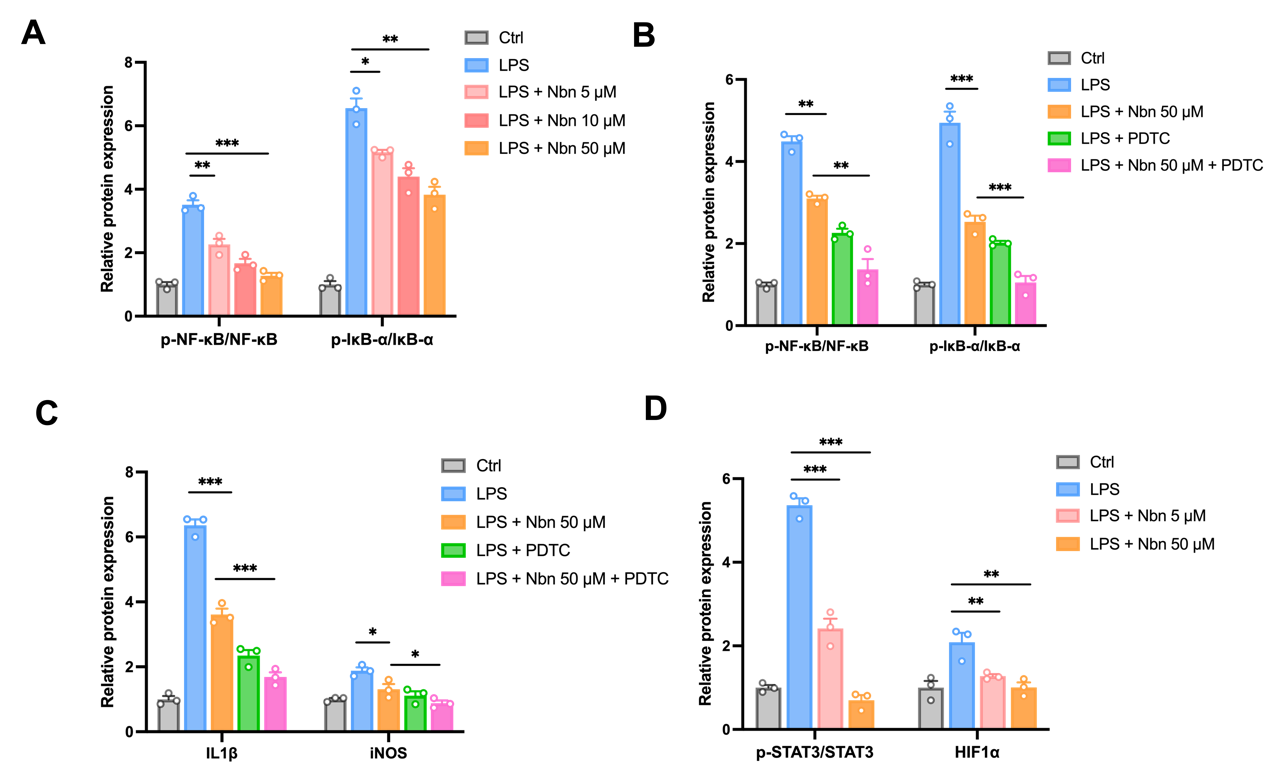


**Figure S4**


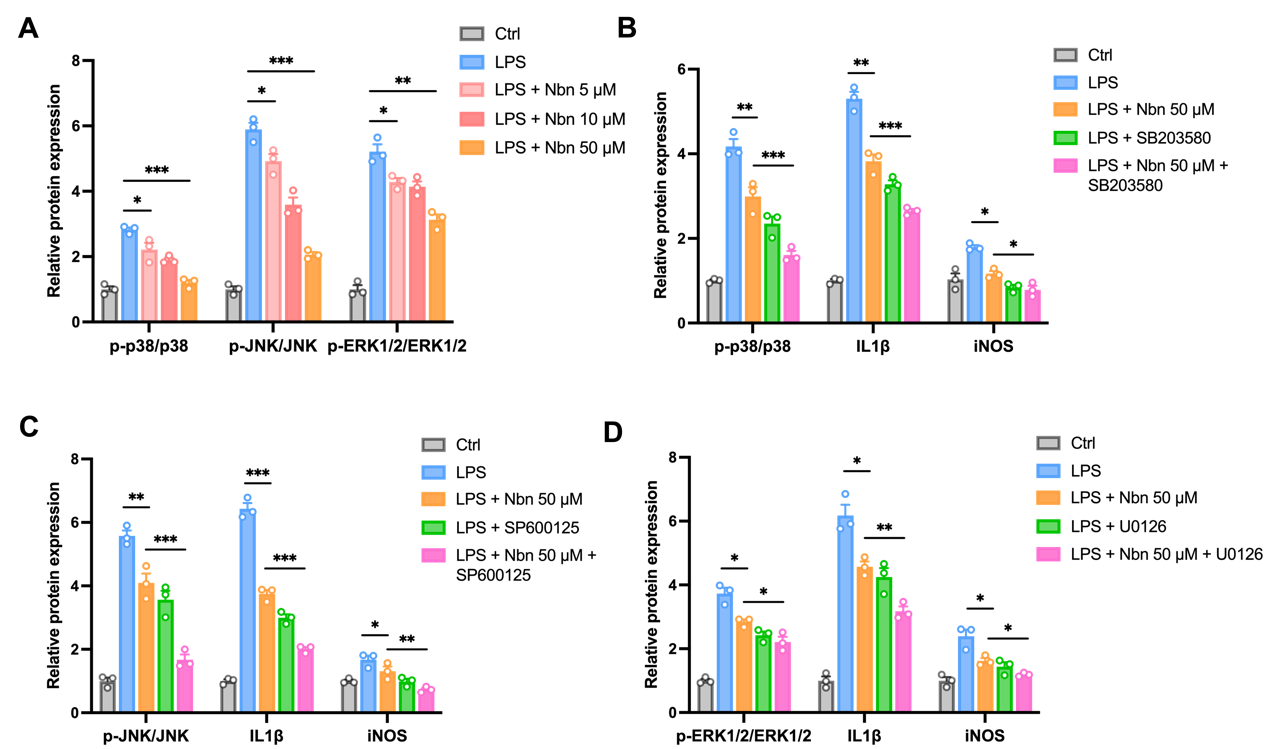


**Figure S5**


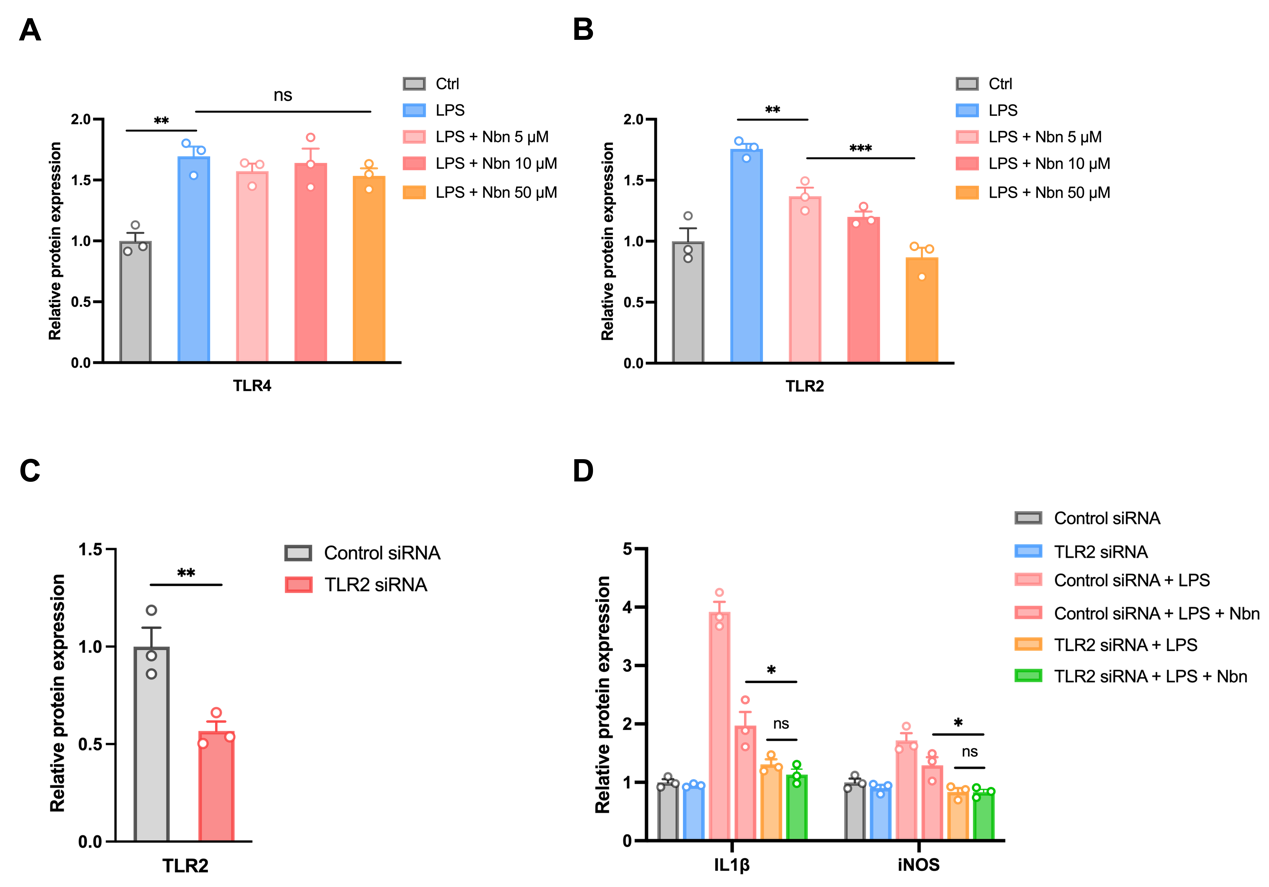


**Figure S6**


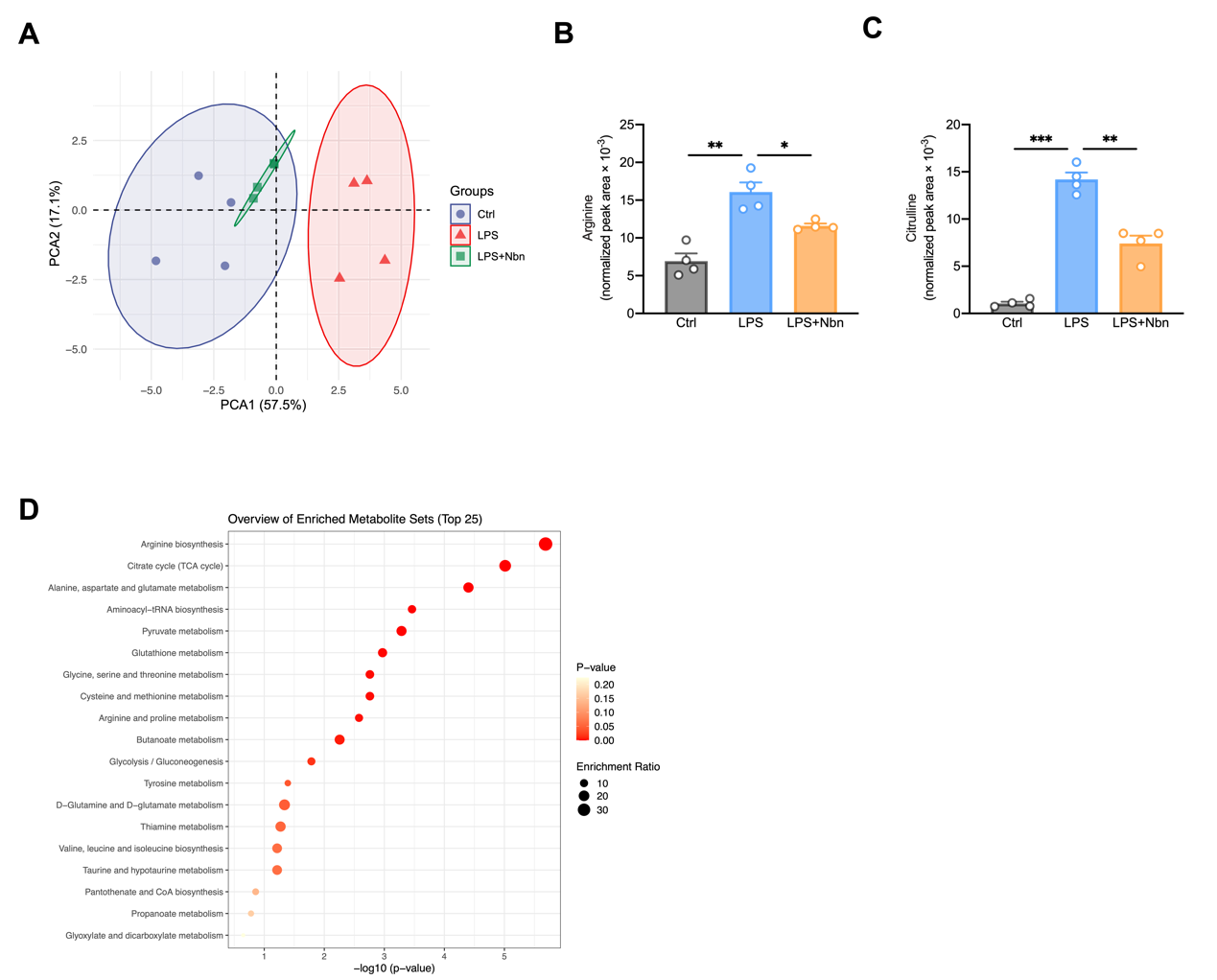

Supplement: Supplementary file 1 [file DataSheet_1.docx]
